# A TAD boundary is preserved upon deletion of the CTCF-rich *Firre* locus

**DOI:** 10.1038/s41467-018-03614-0

**Published:** 2018-04-13

**Authors:** A. Rasim Barutcu, Philipp G. Maass, Jordan P. Lewandowski, Catherine L. Weiner, John L. Rinn

**Affiliations:** 1 Department of Stem Cell and Regenerative Biology, Harvard University, Cambridge, MA 02138 USA; 2 Broad Institute of Massachusetts Institute of Technology and Harvard, Cambridge, MA 02142 USA; 3 Department of Pathology, Beth Israel Deaconess Medical Center, Boston, MA 02215 USA; 4 Department of Molecular and Cellular Biology, Harvard University, Cambridge, MA 02138 USA; 5 Department of Biochemistry, University of Colorado, BioFrontiers Institute, Boulder, CO 80301 USA

## Abstract

The binding of the transcriptional regulator CTCF to the genome has been implicated in the formation of topologically associated domains (TADs). However, the general mechanisms of folding the genome into TADs are not fully understood. Here we test the effects of deleting a CTCF-rich locus on TAD boundary formation. Using genome-wide chromosome conformation capture (Hi-C), we focus on one TAD boundary on chromosome X harboring ~ 15 CTCF binding sites and located at the long non-coding RNA (lncRNA) locus *Firre*. Specifically, this TAD boundary is invariant across evolution, tissues, and temporal dynamics of X-chromosome inactivation. We demonstrate that neither the deletion of this locus nor the ectopic insertion of *Firre* cDNA or its ectopic expression are sufficient to alter TADs in a sex-specific or allele-specific manner. In contrast, *Firre’s* deletion disrupts the chromatin super-loop formation of the inactive X-chromosome. Collectively, our findings suggest that apart from CTCF binding, additional mechanisms may play roles in establishing TAD boundary formation.

## Abstract (web summary)

Although CTCF binding has been implicated in the formation of topologically associated domains (TADs) the mechanisms folding the genome into TADs are not fully understood. Here the authors investigate the TAD boundary on lncRNA locus *Firre*, which has ~ 15 CTCF binding sites, and its organization.

## Introduction

Topologically associated domains (TADs) are units of chromosomes that are separated by regions known as TAD boundaries and exhibit higher frequency of physical contacts between genes and their cognate regulatory elements^1,2^. The organization of the genome into TADs is critical for coordinated transcriptional regulation, chromatin states, and DNA replication^2–4^. The CTCF protein has been identified as one master organizer of this process^5,6^ and its orientation-dependent DNA binding has been implicated in establishing TAD boundaries^7–15^. However, several studies have yielded inconsistent results on the role of CTCF in TAD boundary formation. The depletion of CTCF affects cell survival and leads to global loss of TADs^16^. Although the deletion of a single CTCF site or of a minimal genomic region is sufficient to perturb a TAD boundary in some studies^9,17,18^; others reported that disruptions of a TAD boundary occur only upon deleting very large genomic regions (e.g., 200–400 kb)^1,18^.

To address this conundrum, we focused on a locus on chromosome X that exhibits dense CTCF binding, and that harbors the *Firre* long non-coding RNA (lncRNA)^19,20^. The *Firre* locus topology is evolutionarily conserved across human and mouse, and displays enriched CTCF binding across many cell types^19,20^. More recently, *Firre* has been found to interact with the DXZ4 macrosatellite, a region located at the hinge of the mega-domain formation on the inactive X chromosome (Xi)^12,21,22^. On Xi, DXZ4 is at the anchor of a conserved super-loop formation involving the *Firre* locus, the inactive-X CTCF-binding contact element (ICCE), and a region termed “x75”^23–25^. Although abolishing the DXZ4-*Firre* interaction by DXZ4 deletion does not perturb the X chromosome inactivation process^23^, a DXZ4 inversion results in altered chromatin interaction profiles along the Xi^26^.

In this study, we demonstrate that the *Firre* locus is consistently located at a TAD boundary in multiple species and cell types and we confirm enriched CTCF binding. Based on these features, the *Firre* locus is an ideal candidate to test the role of CTCF binding in TAD boundary formation. Furthermore, in addition to the role of CTCF, using the *Firre* lncRNA locus as a model has the unique advantage of allowing us to test whether the presence and expression of a functional lncRNA contribute to TAD boundary formation, as it was recently proposed^27–32^. Thus, we generated *Firre* deletion and transgenic models in mouse embryonic fibroblasts (MEFs) and embryonic stem cells (mESCs), and performed Hi-C in each of the genetically defined cell types, as well as the corresponding wild-type controls. Interestingly, deletion of *Firre* lncRNA locus, which contains ~ 15 CTCF sites, does not have any effect on TAD boundary formation. Moreover, neither the ectopic insertions of *Firre* complementary DNA (cDNA) nor its inducible expression lead to disruption of existing, or emergence of novel TAD boundaries. In contrast, the Xi super-loop interactions were disrupted upon deleting the CTCF dense *Firre* locus, thereby proposing a role for *Firre* in Xi architecture.

Collectively, our results suggest that in addition to CTCF binding, other mechanisms may be required for proper TAD boundary organization.

## Results

### *Firre* harbors dense CTCF binding and strong TAD boundaries

**Fig. 1:**
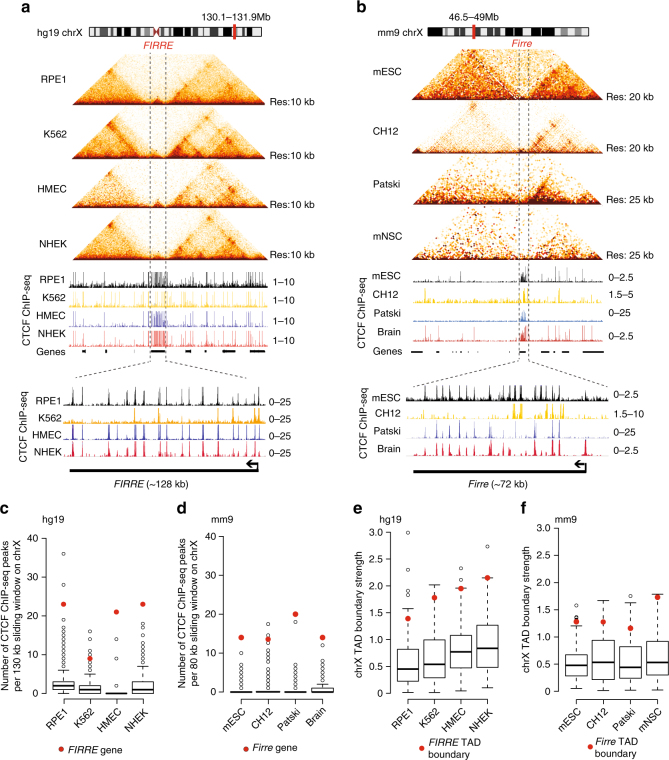
*Firre* is consistently located at a TAD boundary and harbors several CTCF sites. **a** Human Hi-C heatmaps showing ± 1 Mb of the *FIRRE* locus in RPE-1 (female), K562 (female), HMEC (male), and NHEK (unisex) cell lines (upper panel), and CTCF ChIP-seq signals across the *Firre* locus (lower panel). **b** Mouse Hi-C heatmaps depicting the *Firre* locus in mESCs (male), CH12 (female), Patski (female), and mouse neuronal stem cells (mNSCs) (unknown sex, upper panel), and a zoom-in of the CTCF ChIP-seq signal (lower panel). **c**, **d** Box plot showing the number of CTCF peaks for each sliding window on the (**c**) human and (**d**) mouse chromosome X. The bin containing the human and mouse *Firre* genes is shown with a red dot. **e**, **f** Boxplot showing the TAD boundary scores for all the boundaries on the X chromosome in different cell lines in (**e**) human and in (**f**) mouse. The TAD boundary that contains *Firre* is shown with a red dot. Error bars: s.d.

In order to elucidate the contributions of CTCF binding, the genomic insertions, and the expression of a lncRNA to form TAD boundaries, one would ideally interrogate a locus at which all three could be perturbed in parallel. By analyzing publicly available chromatin immunoprecipitation sequencing (ChIP-seq) and Hi-C datasets, we determined that the genomic region around the *Firre* locus harbors one of the highest densities of CTCF binding on chromosome X across multiple cell types (Fig. 1a,b). On average, the *Firre* gene body harbors ~ 15 CTCF sites and extensive CTCF binding occurs across multiple cell types (Fig. 1a,b). We also find that a TAD boundary is directly located at the *Firre* locus (hereinafter referred to as “*Firre* TAD boundary”) (Supplementary Figure 1). This *Firre* TAD boundary is characterized as one of the strongest TAD boundaries on the X-chromosome (Fig. 1c–f, see Methods), and it is consistently stable across human and mouse cell types. These characteristics of the *Firre* locus, together with its evolutionary conservation, makes it an optimal model to test the role of CTCF in local TAD structure.

### Deletion of *Firre* leads to depletion of CTCF binding

**Fig. 2:**
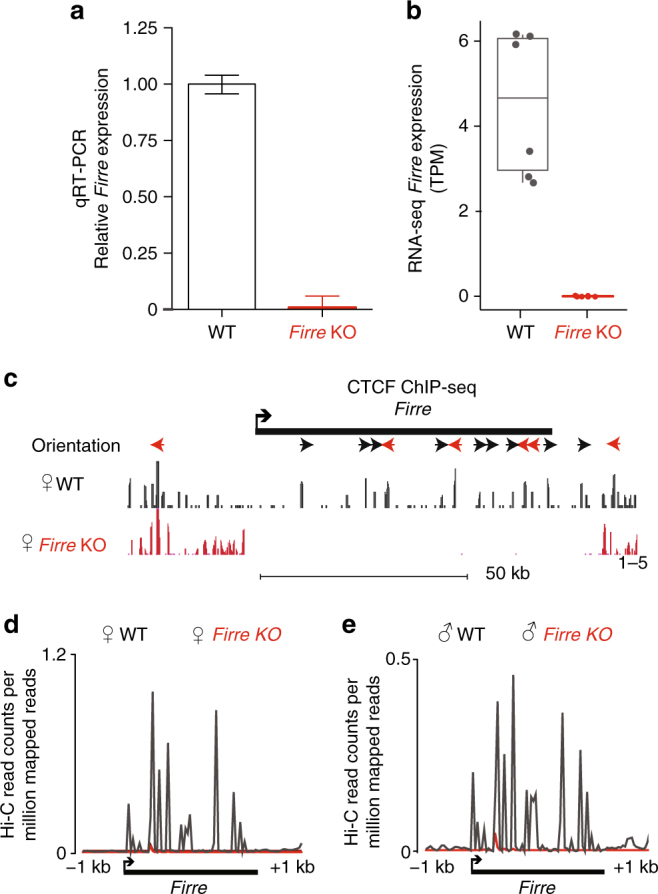
Validation of *Firre* knockout in MEFs. **a** qRT-PCR analysis of *Firre* expression in wild-type and knockout MEFs. Error bars: s.e.m. **b** Plot showing transcripts per million (TPM) values for wild-type and *Firre* KO MEF RNA-seq. Error bars: s.d. **c** CTCF ChIP-seq signal tracks showing the complete loss of CTCF binding at the *Firre* locus in *Firre* KO MEFs (mm9, chrX:47.8–49 Mb). **d**, **e** Hi-C reads per million (RPM) values for the *Firre* locus in (**e**) wild-type and (**f**) *Firre* KO MEFs

To determine whether CTCF binding at the *Firre* locus is necessary for the integrity of the *Firre* TAD boundary, we generated *Firre* knockout (KO) MEFs, in which the ~ 82 kb deletion encompassed the entire *Firre* lncRNA locus, as well as the 3′- and 5′-end (e.g., promoter) regions (Supplementary Figure 2, see Methods). We verified the absence of transcriptional activity of *Firre* by quantitative reverse-transcriptase PCR (qRT-PCR) (Fig. 2a) and RNA sequencing (RNA-seq) (Fig. 2b). The correlation analysis of the RNA-seq replicates showed high reproducibility (Pearson’s correlation > 0.9) in male and female samples (Supplementary Figure 3).

Moreover, we verified the loss of CTCF binding at the deleted *Firre* locus by performing CTCF ChIP-seq in wild-type and *Firre* KO female MEFs. As expected, the CTCF binding encompassing the *Firre* deletion was absent in *Firre* KO MEFs, whereas ChIP-seq profiles of the regions surrounding the deletion were identical to wild-type cells (Fig. 2c). Altogether, we achieved a complete loss of one of the densest CTCF binding regions on the X-chromosome, which allows us to examine their effects on the *Firre* TAD boundary.

### *Firre* deletion leads to preservation of the TAD boundary

We next assessed whether deletion of the *Firre* locus impacted the *Firre* TAD boundary by performing Hi-C in male and female wild-type and *Firre* KO MEFs. Mapping the Hi-C reads to *Firre* further validates its deletion and the Hi-C interaction heatmaps showed high reproducibility (Fig. 2d,e, Supplementary Figure 4–5, and Supplementary Table 1). Importantly, we identify that the *Firre* TAD boundary is precisely located within the *Firre* gene body (Supplementary Figure 7), and resides within the deletion construct (Supplementary Figure 2). To determine whether the effect of CTCF removal to TAD formation is allele specific, we also generated C57BL6/Castaneous (CAST) hybrid MEFs. These hybrid MEFs, which exhibit random Xi^33^, contain one copy of the C57BL6 allele harboring the *Firre* KO allele and one copy of the wild-type CAST allele, and thus permit to distinguish between the parental KO alleles and examine their individual roles on the *Firre* TAD boundary formation in female cells.

**Fig. 3:**
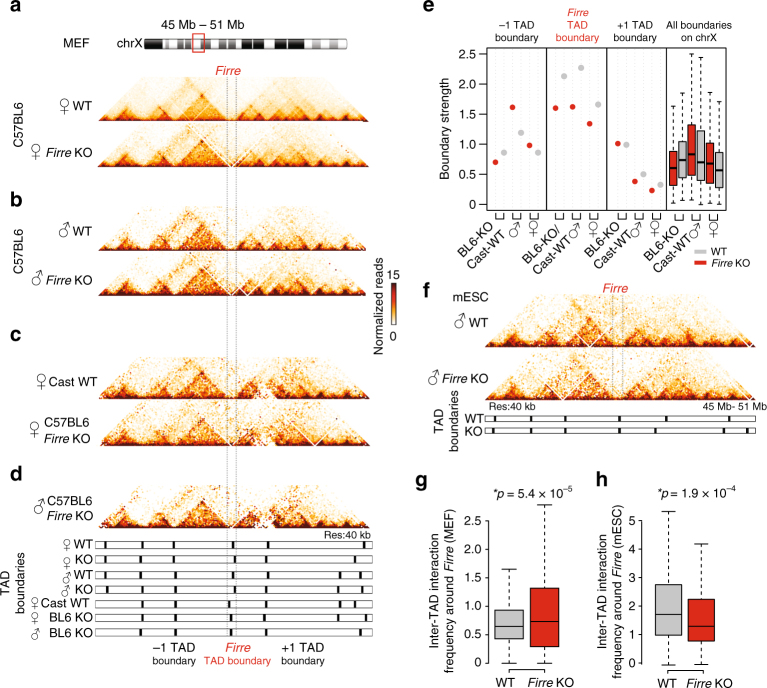
*Firre* KO does not result in disruption of TAD boundaries. **a-d** Hi-C heatmaps showing ± 5 Mb of the *Firre* gene locus (mm9, chr.X: 45–51 Mb) in female wild-type and *Firre* KO MEFs, (**b**) male wild-type and *Firre* KO MEFs, (**c**) allele-specific haploid chromosomes for female Cast (wild type) and C57BL6 (*Firre* KO), and (**d**) male C57BL6 (*Firre* KO). The TAD boundaries and the insulation plot for each Hi-C dataset is depicted below. **e** Dot plots showing the boundary strength of the *Firre*-centered and the neighboring TAD boundaries in wildtype (gray) and *Firre* KO (red) samples. The TAD insulation scores of all TAD boundaries on chromosome X is shown as boxplots on the right panel. **f** Hi-C heatmaps from wild-type (grown on 2i) and *Firre* KO mouse embryonic stem cells (mESCs) (grown on feeders + 2i) showing ± 5 Mb of the *Firre* locus. **g**, **h** Boxplot showing the inter-TAD interaction frequency between the TAD domains neighboring the *Firre* locus in wild-type (gray) and *Firre* KO (red) cells (**g**) in female MEFs and (**h**) in mESCs. Error bars: s.d. (*p-value: Wilcoxon rank-sum test)

Remarkably, despite the removal of one of the highest CTCF-binding densities and the complete deletion of the *Firre* lncRNA on chromosome X (Fig. 1), the TAD boundary is consistently preserved in either male, female, or hybrid MEFs (Fig. 3a–d). Although the insulation scores are decreased at the *Firre* TAD boundary in the KO samples compared with controls, neither the *Firre* nor the neighboring TAD boundaries display any significant changes in their insulation profiles (Fig. 3e). To rule out that these results may be cell-type specific, we repeated Hi-C in wild-type and *Firre* KO male mouse embryonic stem cells (mESCs) grown on feeder cells (Supplementary Figure 6, see Methods). Consistent with the MEF Hi-C results, the *Firre* TAD boundary is also preserved in *Firre* KO mESCs. (Fig. 3f). By calculating the inter-TAD interaction frequency in wild-type and *Firre* KO MEFs and mESCs, we observe that the TADs surrounding *Firre* exhibit a higher interaction frequency in the *Firre* KO MEFs (Fig. 3g), but not in *Firre* KO mESCs (Fig. 3h), likely to be due to cell-type-specific differences. This finding in MEFs suggests that even though the *Firre* TAD boundary is preserved, it has been weakened by the genetic deletion, allowing a higher rate of interactions overpassing the boundary in *Firre* KO MEFs when compared with wild-type controls.

Next, to assess whether the CTCF sites surrounding *Firre* deletion may act as insulators that could preserve the TAD boundary upon *Firre* KO, we examined the CTCF ChIP-seq data from female MEFs (Fig. 2), as well as publicly available datasets, for the regions flanking the *Firre* deletion across several cell types. We identified four peaks flanking the *Firre* gene body on both sides (Supplementary Figure 7). Of note, these surrounding CTCF peaks are outside of the *Firre* TAD boundary that is located within *Firre* gene body (Supplementary Figure 7). By analyzing publicly available ChIP-seq datasets, we observed that C2C12 cells display reduced binding at the surrounding CTCF sites (Supplementary Figure 8a–c). We reasoned that if the *Firre* TAD boundary was present in C2C12 cells with minimal CTCF binding at these sites, it would suggest that the surrounding CTCF sites are not essential for the preservation of the *Firre* TAD boundary. To determine whether the surrounding CTCF sites are required in establishing the *Firre* TAD boundary, we performed Hi-C in a third cell line: C2C12 murine muscle cells. We identified a robust TAD boundary formation at the *Firre* locus in this cell line that displays reduced binding at the surrounding CTCF sites (Supplementary Figure 8a–d). Together, these analyses and experiments suggest that the more variable surrounding CTCF sites can be dispensable for the preservation of the *Firre* TAD boundary (Supplementary Figure 8d, see Discussion).

Altogether, our findings indicate that the formation and maintenance of the conserved *Firre* TAD boundary is resilient to (i) removal of CTCF binding, (ii) deletion of the *Firre* locus, (iii) loss of *Firre* transcription, and (iv) absence of *Firre’s* functional lncRNA transcript.

### *Firre* cDNA insertions do not alter TAD boundaries

**Fig. 4:**
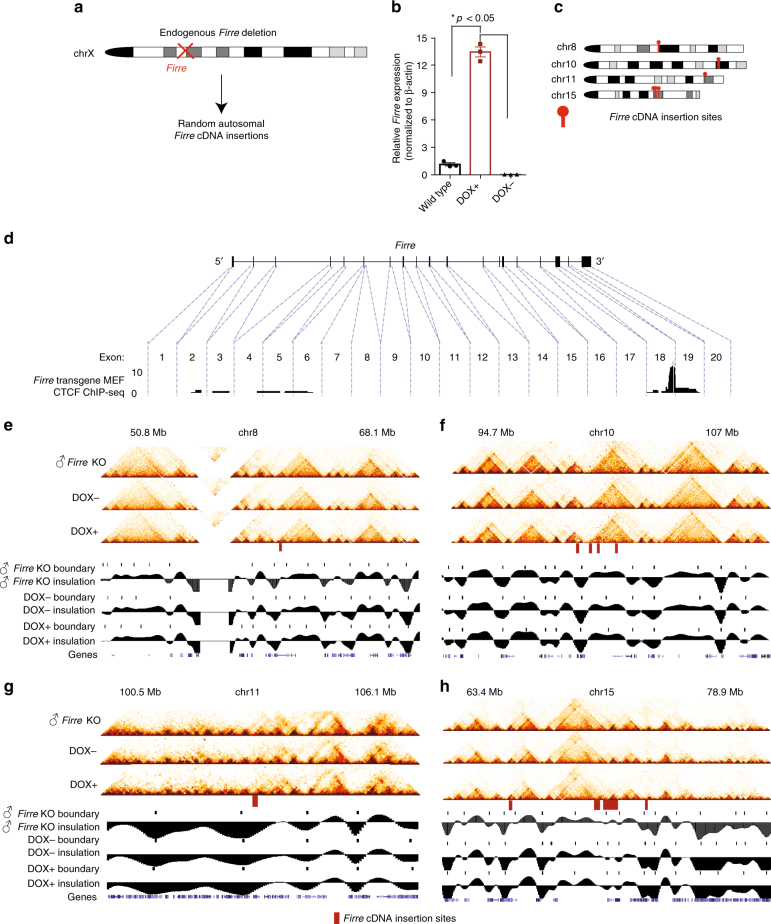
TAD boundaries are preserved upon ectopic *Firre* cDNA insertion and its induced expression at target sites. **a** Cartoon depicting the approach for the generation of the transgenic MEFs with endogeous *Firre* KO and ectopic *Firre* cDNA insertions. **b** qRT-PCR showing the induced expression of the *Firre* cDNA in wild type, DOX^−^, and DOX^+^ conditions. Error bars: s.e.m. (*p-value: *t*-test). **c** Chromosome ideograms showing the *Firre* cDNA insertion sites on four different chromosomes. **d** CTCF ChIP-seq signal from DOX^−^ transgenic MEFs for each of the exons of *Firre* cDNA at randomly inserted loci. As the transgenic MEFs harbor an endogenous *Firre* deletion, the intronic regions did not harbor any ChIP-seq signal. **e-h** Hi-C heatmaps showing the TAD organization, TAD boundary position, and the insulation plots for male *Firre* KO, DOX^−^, and DOX^+^ samples ± ~ 5 Mb of *Firre* cDNA insertion sites on (**e**) chr 8, (**f**) chr 10, (**g**) chr 11, and (**h**) chr 15

It was previously hypothesized that apart from binding of CTCF, the presence of lncRNAs, or their expression, may be a driving factor to establish TAD boundaries^29,32^. Therefore, although the deletion of the CTCF-rich *Firre* locus is not sufficient to disrupt TAD boundary formation, we sought to determine whether an ectopically inserted *Firre* cDNA would establish novel TAD boundaries, either in the presence or absence of its transcription. To do this, we inserted *Firre* cDNA under the regulation of a doxycycline (DOX)-inducible promoter into random genomic sites in male MEFs that harbor one active X chromosome, but with an endogenous *Firre* deletion (Fig. 4a). Upon DOX induction (DOX^+^), we measured a ~ 13-fold increase of *Firre* cDNA expression from the ectopic loci by qRT-PCR, confirming the induced expression of the ectopically inserted *Firre* cDNA (Fig. 4b). We then performed Hi-C on non-induced (DOX^−^) and induced (DOX^+^) transgenic MEFs, and identified four chromosomes with multiple *Firre* insertions (Fig. 4c, Methods). To determine whether the inserted *Firre* cDNA can recruit CTCF, we performed CTCF ChIP-seq on DOX^−^ transgenic MEFs and found CTCF binding on exon 18 on the *Firre* cDNA (Fig. 4d, introns and intronic CTCF binding were excluded).

We next tested whether the local TAD boundaries at the ectopic insertion sites were altered in any way by the presence of the *Firre* cDNA or ectopic CTCF binding. By comparing the DOX^−^ transgenic *Firre* MEFs with male *Firre* KO MEFs, we found that none of the ectopic *Firre* insertions, despite active CTCF binding, significantly altered the local genomic structure or created new TAD boundaries (Fig. 4e–h). We then tested whether the transcriptional activation of *Firre* at these ectopic loci would have an impact on TAD organization. To do this, we performed Hi-C on DOX^+^ transgenic MEFs, and compared the TAD boundaries with Hi-C data of DOX^−^ transgenic MEFs. We observed that the presence of transcription at the *Firre* insertion sites did not affect TAD boundary formation (Fig. 4e–h). Together, these data suggest that neither ectopically inserted CTCF sites nor the ectopic genomic insertion of a cDNA, nor the act of transcription at the ectopically inserted loci are sufficient to alter endogenous TAD boundaries or create novel TADs at target sites.

### *Firre* deletion results in loss of *Firre*-DXZ4 interactions

**Fig. 5:**
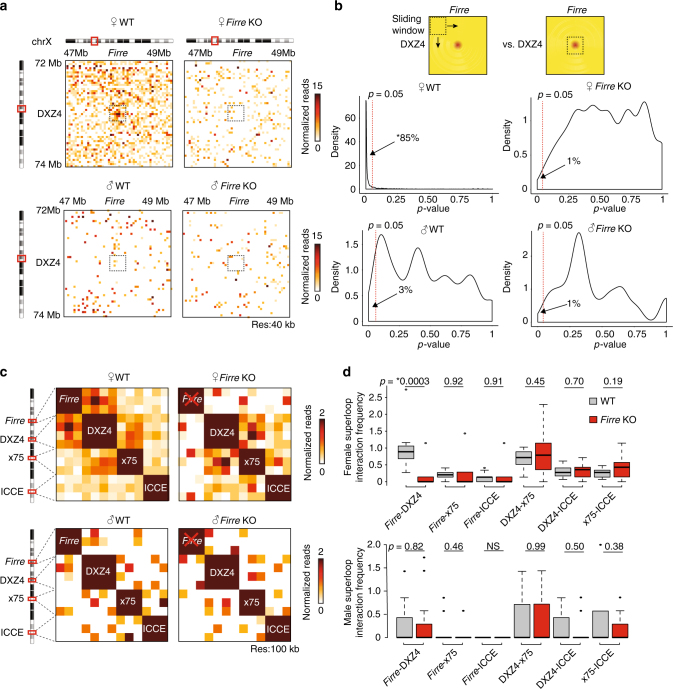
*Firre* KO results in a loss of *Firre*-DXZ4, and changes in super-loop interactions in female MEFs. **a** Hi-C heatmaps at 40 kb resolution in male and female wildtype and *Firre* KO MEFs showing the interaction frequency between *Firre* and DXZ4 ± 1 Mb. **b** Forty-two windows of 40 kb bins were slid across the entire heatmap (2 Mb × 2 Mb in size) to compare the interaction frequency at each position of female and male WT versus KO conditions to the *Firre*-DXZ4 interaction by *t*-tests. The *p*-value distribution in the female or male wild-type samples indicate a drastically enriched significance of *Firre*-DXZ4 interactions when compared with either the female *Firre* KO or male samples (*p < 0.05, one-way ANOVA). **c** Hi-C heatmap at 100 kb resolution showing the zoomed-in interactions between the mouse *Firre*, DXZ4, x75, and ICCE regions ± 100 kb in female and male wild-type and *Firre* KO MEFs. **d** Boxplots showing the interactions among the super-loop regions in wild-type (gray) and *Firre* KO (red) female (top) and male (bottom) MEF samples. The sample sizes of the boxplots are *n* = 12 for *Firre*-DXZ4, DXZ4-x75, and DXZ4-ICCE interactions, and *n* = 9 for all other combinations. *p*-value: *t*-test. Error bars: s.d

X chromosome inactivation is a critical biological process that involves massive reorganization of chromosome X with loss of local structures and formation of transcriptionally silent mega-domains^12,21–25^. The DXZ4 macrosatellite, which is located at the hinge point of two mega-domains, has been shown to interact with the *Firre* locus^23^. We therefore investigated the role of *Firre* in this interaction by Hi-C. In female primary MEFs, we confirmed the previous finding that *Firre* strongly interacted with DXZ4. However, this interaction was abolished in female *Firre* KO MEFs (Fig. 5a). In contrast, *Firre* did not show a specific association with DXZ4 in male MEFs, in which the only X chromosome is active (Fig. 5a). To validate the specificity of the *Firre*-DXZ4 interaction, we selected a window on the heatmap (6 × 7 = 42 40 kb bins) and slid it bin-by-bin across the entire heatmap (2 Mb x 2 Mb in size). We then compared the interaction frequency of the sliding window at each position with the detected *Firre*-DXZ4 interaction (same window size) via a *t*-test. We performed this analysis for each condition (female and male wildtype and *Firre* KO). By plotting the density of the *p*-values (*t*-test), we observed in the female wild-type dataset that the *Firre*-DXZ4 interaction is significantly enriched compared with the female *Firre* KO and the male samples (*p* < 0.05, one-way analysis of variance).

In human cells, the interactions of *Firre* on Xi have been reported to be associated with the formation of a super-loop involving the DXZ4 macrosatellite, x75, and ICCE regions, all of which are lncRNA loci^23^. To investigate whether such a super-loop might occur in mice, and to determine if *Firre* is required to form any such structure, we investigated the interactions between mouse *Firre*, DXZ4, x75, and ICCE regions (Fig. 5c). Hi-C in female *Firre* KO MEFs showed that *Firre* deletion disrupted its association with DXZ4 (Fig. 5c,d). In contrast, male wildtype and KO MEFs did not display any notable changes (Fig. 5c,d). These results suggest an architectural role of *Firre* in the Xi super-loop formation.

**Fig. 6:**
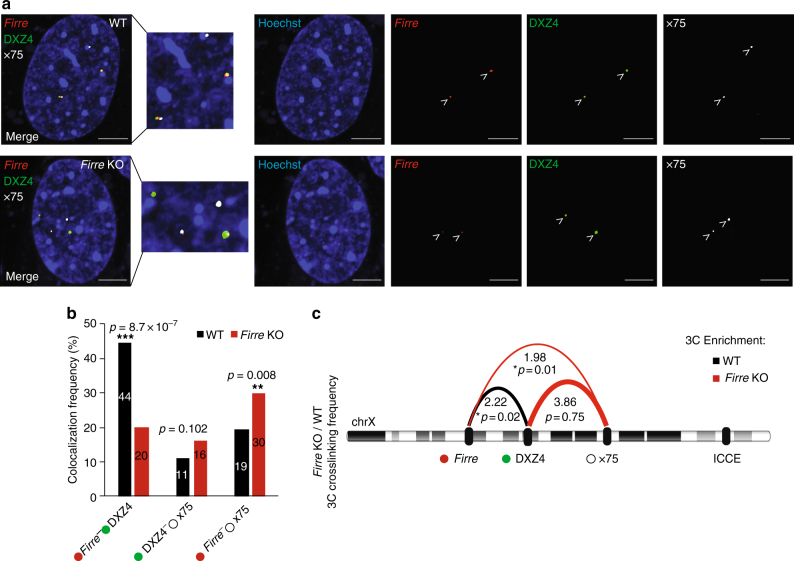
CRISPR live-cell imaging and 3C validates the changes in super-loop interactions. **a** Four-color CRISPR live-cell imaging (CLING) from female wild-type and *Firre* KO MEFs. *Firre* (red), DXZ4 (green), and x75 (white) loci were simultaneously visualized with Hoechst staining (blue). Pseudo-coloring was used for visual simplicity. Scale bar: 5 μm. **b** Quantification of the colocalization percentages between *Firre*-DXZ4, DXZ4-x75, and *Firre*-x75 between wild-type (black) and *Firre* KO (red) MEFs. (*p-value: *χ*^2^-test, *n* > 80 nuclei). Error bars: s.e.m. **c** Chromosome conformation capture (3C) analysis showing the interaction frequency ratios of *Firre*-DXZ4, DXZ4-x75, and *Firre*-x75 in female *Firre* KO vs. wild-type MEFs (*p-value: *t*-test, *n* = 3). The black arc indicates 3C enrichment in the wild-type samples, whereas the red arc represents enrichment in the *Firre* KO samples

To validate the loss of *Firre*-DXZ4-x75 interactions, we performed four-color CRISPR/Cas9 live-cell imaging (CLING) in female wild-type and *Firre* KO MEFs^34^. To do this, we used a nuclease-null mutant of the *Streptococcus pyogenes* Cas9 protein (dCas9) and pools of three single-guide RNAs (sgRNAs) separately targeting *Firre*, DXZ4, and x75 regions (Supplementary Table 4). The sgRNAs targeting *Firre* and DXZ4 are internally appended with three copies of MS2 and PP7 motifs, respectively, and the x75 sgRNAs with six copies of Puf1 RNA-aptamer motifs. Co-transfection of corresponding RNA-binding proteins (MS2, PP7, or Pum1) fused to a fluorescent proteins (mVenus, mCherry, or iRFP670), in combination with nuclear Hoechst staining, has enabled us to simultaneously visualize *Firre*, DXZ4, and x75 loci in living cells^34^. The sgRNAs targeting the *Firre* locus were designed within 650 bp adjacent to the *Firre* deletion (5′-end), so that both the wild-type and *Firre* KO loci could be visualized. When we quantified the colocalization frequencies, we detected a significant reduction between *Firre* and DXZ4 in *Firre* KO cells, when compared with wild-type controls (*p* = 8.7 × 10^−7^, *χ*^2^-test, Fig. 6a,b). In contrast, the *Firre* KO MEFs displayed a modest, nonsignificant colocalization increase for the DXZ4-x75 interaction (*p* = 0.102, *χ*^2^-test) when compared with wild-type MEFs. Of note, we observed a significantly increased colocalization of *Firre* with the x75 region in KO MEFs (*p* = 0.008, *χ*^2^-test, Fig. 6a,b).

To further verify the changes in the super-loop formation in the *Firre* KO cells, we performed chromosome conformation capture^35^ (3C) in female wild-type and *Firre* KO MEFs. We confirmed the significant loss of *Firre*-DXZ4 (*p* = 0.02, *t*-test) in the *Firre* KO MEFs when compared with wild-type MEFs (Fig. 6c and Supplementary Figure 9). The difference between the DXZ4-x75 3C interaction frequency correlated with Hi-C and live-cell imaging, although it was not significant (*p* = 0.75, *t*-test). Consistent with the live-cell imaging results, the *Firre*-x75 3C interaction frequency was also significantly increased (*p* = 0.01, *t*-test) in the *Firre* KO sample (Fig. 6c). Taken together, through Hi-C, 3C, and CLING experiments, we conclude that the *Firre* locus and/or its lncRNA product are important in regulating super-loop interactions in female cells.

## Discussion

The genome harbors a non-random three-dimensional conformation that is dynamically reconfigured. The organization of one of the fundamental units, TAD structures, is strongly associated with directional binding of CTCF^8^. The current model explaining the TAD boundaries, known as the loop extrusion model, suggests that loop extruding factors such as cohesin and condensin, slide along the chromatin and protrude the DNA until two convergent CTCF sites are reached^8,14,36^, therefore implicating CTCF as one of the important organizers of chromatin^7,13,37^. However, discrepancies have been reported in terms of the requirement of deleting CTCF-binding sites, or depleting CTCF to disrupt TAD boundaries^9,11,16,38,39^.

In this study, we genetically removed the evolutionary conserved *Firre* locus that has both one of the highest CTCF densities in the genome across and a stable TAD boundary across many cell types and species. Interestingly, the *Firre* KO did not lead to perturbation of the TAD boundary, but led to a decrease in the strength of the *Firre* TAD boundary in MEFs, suggesting that TAD organization at the *Firre* locus may not be individually dependent on CTCF binding or *Firre* lncRNA expression. Although not directly located at the *Firre* TAD boundary (Supplementary Figure 7), additional flanking CTCF sites outside of the *Firre* deletion may compensate for the loss of CTCF binding at this locus and keep *Firre*’s TAD boundary formation intact in *Firre* KO cells. In addition, we further show that, despite the recruitment of CTCF, the ectopic insertion or the induced expression of the *Firre* cDNA at ectopic sites are not sufficient to alter the TAD landscape of local chromatin environment. These results suggest that the establishment of TADs may be mediated by CTCF-independent, multi-layered mechanisms, that are likely to be redundant to preserve the robustness of TAD structures. The current mechanistic model for intra-chromosomal chromatin organization is the loop extrusion model^7,13,37^. Consistent with this model, the inversion of CTCF sites abrogates chromatin looping interactions^8,36^, and the depletion of CTCF results in the weakening of TAD boundaries^16^. In addition, cohesin removal also leads to the loss of TADs^40,41^. The *Firre* locus, which is bound by CTCF, is also extensively bound by cohesin, as well as the transcription factor YY1, which was identified to associate with CTCF^42^ and was implicated as a structural regulator of looping interactions^43^. The preservation of the *Firre* TAD boundary in our deletion model therefore suggest that these two factors may also harbor redundant functions.

We therefore conclude that, in addition to the well-established mechanism of convergent CTCF sites being the primary factor for loop extrusion, alternative mechanisms may also be responsible for the establishment of TADs. Several recent studies have provided support to this hypothesis^8,44,45^.

In addition to convergent CTCF sites, there are other aspects of chromatin which can mediate TAD boundary formation. It has been shown that even though ~ 75 % of all looping interactions can be explained by convergent CTCF orientations, the remaining ~ 25 % of interactions were bound by CTCF with forward–forward, reverse–reverse, and divergently oriented CTCF motifs^8^. In another report, ~ 10 % of all the CTCF motifs at the anchor of loops did not obey the convergent rule^45^. Interestingly, the folding of *Drosophila* contact domains was found to be independent of CTCF orientation^44^, suggesting the role of alternative architectural proteins in chromosome structure. Consistent with this, in human cells, *FIRRE* appears to form a circular structure with divergently oriented CTCF sites, whereas this circular structure is not prevalent in mouse (Supplementary Figure 10–11). Importantly, in the mouse genome, the CTCF orientations at the 3′-end of *Firre* gene and the neighboring boundary (the − 1 TAD boundary in Fig. 3a) show forward–forward CTCF motif orientations, suggesting the presence of interactions with non-convergent CTCF-binding motifs (Supplementary Figure 11).

There is further evidence in the literature for CTCF-independent mechanisms of TAD boundary formation. For instance, gene activation and repression was shown to have important roles in genome folding independent of CTCF^44^. Additional studies have also highlighted the role of chromatin condensation and de-condensation via topoisomerases and chromatin remodeling factors to organize the TAD structures^46–50^, and the stiffness of chromatin has been postulated to mediate TAD boundary formation^51^. Altogether, our findings therefore provide experimental insight into the redundancy of CTCF in TAD boundary organization, in the context of the *Firre* locus.

Through Hi-C, CLING, and 3C, we furthermore show that the deletion of *Firre* results in the perturbation of super-loop interactions, specifically with the DXZ4 macrosatellite (Figs. 5,6). Interestingly, in female *Firre* KO MEFs, via CLING and 3C, we identified an increased interaction frequency between the region surrounding *Firre* and x75, suggesting that apart from the lncRNA genes in these loci, other features of chromatin may also be involved in mediating the super-loop formation. Future studies will determine the functional significance of the disruptions of the super-loop formation on X chromosome inactivation.

In summary, our study suggests the redundancy of CTCF binding, local lncRNA, and its transcription in the establishment of TADs, and sheds new light on an architectural role for *Firre* in higher order X chromosome architecture.

## Methods

### Publicly available Hi-C and CTCF ChIP-seq analysis

Hi-C interaction matrices for K562 (female), HMEC (male), NHEK (unisex), sperm (male), Patski fibroblasts (female), and mouse neuronal stem cells (male) were downloaded using the Juicebox software^12,52,53^, and the heatmaps were generated with the publicly available script “heatmap.pl” available through Github (https://github.com/dekkerlab/cworld-dekker). The RPE-1 (female) and mESC Hi-C datasets^2,23^ were downloaded and analyzed with the HiC-Pro package v2.7.8^54^. The CTCF ChIP-seq datasets for human RPE-1 cells and mouse sperm cells were downloaded from previously published studies^55,56^. All the other CTCF datasets were downloaded from the ENCODE project database^57^. To identify the CTCF enrichment of genomic loci, we calculated the number of CTCF peaks for each 130 kb and 80 kb sliding window on chromosome X, for human and the mouse genomes, respectively.

### Preparation of *Firre* KO cells

The *Firre* KO mouse was generated by inserting a neomycin cassette flanked by loxP sites at position mm9:chrX:47908463-47908464, and by inserting a hygromycin cassette flanked by loxP sites at mm9:chrX:47990293-47990294. Recombination between the loxP sites resulted in a ~ 82 kb deletion around the *Firre* locus. A tet-inducible *Firre* overexpression mouse was generated by cloning a mouse isoform of *Firre* into a modified pTRE2 vector that lacks the beta globin intron and the construct was microinjected into C57BL6/J-129 F1 hybrid embryos in order to generate transgenic mice. MEFs were prepared at E13.5. Embryos were collected in 1 × phosphate-buffered saline (PBS) and individual embryos were eviscerated and the head, forelimbs, and hind limbs were removed. Individual embryo caucuses were then placed into 6 cm^2^ tissue culture plate and 1 mL of pre-warmed 37 °C TrypLE (Thermo Fisher 12604013) was added to each well and incubated for 20 min at 37 °C. Embryos were dissociated using a P1000 tip with gently pipetting and then MEF media was added. Cells were cultured for 5–7 days and cryostocks of individual lines were generated. Subsequent experiments were performed from thaws from the cryostocks^58^. *Firre* transgenic female mice were mated to CAGs-rtTA3 males (Jackson Lab 016532) and individual embryos at E13.5 were collected and used to prepare MEF lines. *Firre* wild-type and KO MEFs were also prepared from E13.5 embryos. MEFs were genotyped for the sex-specific region (Sry), *Firre* wild-type, *Firre* KO, *Firre* transgenic, and rtTA3 alleles (Supplementary Table 2). MEFs were used up to passage 4. MEFs were cultured in 1 × Dulbecco’s modified Eagle’s medium (DMEM) (Invitrogen 11965-118), fetal bovine sSerum (Gibco 10082139), l-glutamine (ThermoFisher 25030081), and penicillin/streptomycin (ThermoFisher 15140122). The DOX induction was performed by treating the cells with 2 µg/mL DOX (Sigma D9891) for 72 h. The media was changed every 48 h.

Wild-type C2C12 cells were obtained from ATCC (CRL-1772) and were cultured by using the same conditions as MEFs described above.

ESCs were derived on MEF, Lif, and one inhibitor (GSK inhibitor) ES media with serum replacement. Freeze downs were at passage 2 from a 24-well plate. The wild-type N3 male mESCs were cultured with 2i media. The *Firre* KO mESCs were co-cultured with irradiated feeder MEFs (ATCC, SCRC-1040.1) with 2i media. Feeder depletion was performed by plating disassociated cells on a gelatinized plate for 20 min at 37 °C followed by reseeding the supernatant containing the mESCs to a new gelatinized plate. This procedure was repeated two consecutive times. The animal protocols in this study have been approved by Institutional Animal Care and Use Committee (IACUC) and Harvard University (11-13-1).

### qRT-PCR analysis

RNA was extracted by using TRIzol Reagent (ThermoFisher 15596018) and cDNA was generated by using the SuperScript III Reverse Transcriptase kit (ThermoFisher 18080044), according to manufacturer’s instructions. qRT-PCR was performed by using the primers listed in Supplementary Table 2. The qRT-PCR data was analyzed by using the 2^(–delta/delta Ct)^ method.

### RNA sequencing

Total RNA was extracted from wild-type and *Firre* KO MEFs using TRIzol followed by RNeasy Mini Qiagen extraction kit according to manufacturer’s protocol. RNA-seq libraries were generated using the Illumina TruSeq (version 2) kit and sequenced on Illumina HiSeq2500 instrument. RNA-seq reads were aligned with RNA-seq analysis performed by filtering and mapping the reads by Bowtie 2^59^, quantifying the transcripts by RSEM v1.2.29^60^. Differential gene expression was calculated using the Deseq2 version 1.4.5 package in R 3.1.0 using the mean value of gene-wise dispersion estimates^61^. To find significant differentially expressed genes, we used < 0.01 for adjusted *p*-value and > 1 log2 fold change.

### ChIP-seq analysis

The ChIP assay was performed by first crosslinking ~ 10 million cells with 1% formaldehyde at room temperature for 10 min, washing the cells twice with 1 × PBS, and then followed by lysing the cells with 1 mL of Lysis Buffer A (50 mM HEPES, 140 nM NaCl, 1 mM EDTA pH 8.0, 10% Glycerol (Sigma G5150), 0.5% NP-40 (Igepal CA-630, Sigma I3021), 0.25% Triton X-100), centrifuging the cells at 1,350 × *g* for 5 min at 4 °C, and re-suspending the pellet with 1 mL of Lysis Buffer B (10 mM Tris-HCl pH 8.0, 200 nM NaCl, 1 mM EDTA pH 8.0, 1 mM EGTA), centrifuging the cells at 1350 × *g* for 5 min at 4 °C, and a final resuspension of the pellet with 300 µL of Lysis Buffer C (10 mM Tris-HCl, pH 8.0, 100 mM NaCl, 1 mM EDTA pH 8.0, 1 mM EGTA, 0.1% Sodium deoxycholate (Sigma D6750), and 0.5% *N*-lauroylsarcosine (Sigma L5777)^62^. All the lysis buffers included the cOmplete, Mini Protease Inhibitor Cocktail (Sigma 11836153001). The chromatin was sheared by using a Covaris S220 instrument. The pull-down was performed using 10 µg of CTCF antibody (1:1000 dilution, Millipore Sigma 07–729). The reads were aligned to the mm9 human genome using the Bowtie2 tool^59^. The ChIP-seq data was analyzed using the HOMER suite^63^. The motif orientations were determined by the FIMO software^64^, using the CTCF motif position weight matrix (MA0139.1) from the JASPAR database^65^.

### Generation of Hi-C libraries

Hi-C libraries were generated with an in-situ ligation protocol using the *HindIII* restriction enzyme^66^, Briefly, ~ 25 million cells were crosslinked with 1% formaldehyde for 10 minutes at room temperature. Then, the chromatin was digested with *HindIII*, end-labeled with biotin-14-dCTP (Thermo Fisher 19519016), and in-situ ligation was performed. Following phenol–chloroform extraction, the biotin was removed from unligated ends and the DNA was sheared by using a Covaris S220 instrument. After A-tailing, biotin pull-down, and adapter ligation, paired-end sequencing was performed on a HiSeq instrument. Each Hi-C library was generated in at least two biological replicates from MEFs prepared from at least two distinct mouse embryos, with the exception of transgenic MEFs and mESCs, which were prepared in technical duplicates.

### Analysis of Hi-C datasets

Hi-C mapping, filtering, correction, and binning was performed with the HiC-Pro software v2.7.8^54^. The reads were mapped to the mm9 mouse reference genome. For allele-specific Hi-C analysis, a high-quality single-nucleotide polymorphism (SNP) list for the C57BL6NJ and CastEiJ genomes was generated by using the mm9 annotation from the Mouse Sanger Database^67^, using the HiC-Pro “extract_snps.py” tool. Next, C57BL6NJ/CastEiJ SNP-masked mm9 reference genome was generated using the bedtools “maskfasta” tool^68^. Then, allele-specific Hi-C data was analyzed using the HiC-Pro “ALLELE_SPECIFIC_SNP” configuration option^54^. Supplementary Table 1 lists the paired-end read counts for each biological replicate of the Hi-C datasets. There was a high correlation among all the Hi-C biological replicates, indicating the high quality and reproducibility of the datasets. Therefore, we pooled all biological replicates for each condition and mapped, filtered, corrected and binned them as a single Hi-C dataset and used the pooled datasets for all subsequent analyses. For the Hi-C analysis of endogenous *Firre KO* with ectopic *Firre* cDNA insertion MEFs, the Hi-C reads were mapped to a custom mm9 genome with an extra chromosome consisting of the insertion cassette (TRE element, cytomegalovirus promoter, *Firre* cDNA, and polyA terminator). Then, “inter-chromosomal interactions” between the *Firre* custom chromosome and all the mouse chromosomes were plotted in the DOX^−^ and DOX^+^ Hi-C datasets. The regions that displayed consistent “interactions” with the *Firre* custom chromosome in DOX^−^ and DOX^+^ samples were determined as *Firre* cDNA insertion sites. For the super-loop analysis in Fig. 5, the human x75 and ICCE coordinates^23^ were lifted over to the mm9 genome. The center line of all boxplots represent the median, the whiskers represent the maximum and minimum values, and the upper and lower bounds of the box represent the top first and third quantiles of the datasets.

### Insulation and TAD Boundary Analysis

TAD analysis was performed with the “Insulation Method”^69^. A publicly available script (matrix2insulation.pl) was used to detect the TAD boundaries, with the following options: “–is 480000 –ids 320000 –im iqrMean –nt 0 –ss 160000 –yb 1.5 –bmoe 0 –bg”. The script can be accessed through GitHub (https://github.com/dekkerlab/cworld-dekker).

### Chromosome conformation capture

3C assay was performed by using the *HindIII* enzyme^35^, with the modification that in-situ ligation was performed^66^. MEFs were fixed with 1% formaldehyde in serum-free α-MEM for 10 min at room temperature. Formaldehyde was quenched by the addition of 0.125 M glycine. Nuclei were released by dounce homogenization in ice-cold lysis buffer (10 mM Tris-HCl pH 8.0, 10 mM NaCl, 0.2% NP-40) containing cOmplete, Mini Protease Inhibitor Cocktail (Sigma 11836153001). Nuclei were collected and subjected to overnight digestion at 37 °C with 400 U of *HindIII* (NEB R0104L). Then, in-situ ligation was performed for 4 h at 16 °C^66^. The crosslinks were reversed by incubating the samples at 65 °C overnight in the presence of proteinase K, and the DNA was purified by phenol–chloroform extraction. Supplementary Table 3 lists the 3C primers used in this study. The 3C libraries were prepared in three biological triplicates, and the 3C amplicons from each replicate were quantified in three technical replicates. The sample to sample variation was normalized by using the *Gapdh* region as a control locus. The results in Fig. 6 represent the 3C interaction frequency ratios of *Firre* KO / wildtype values. All 3C products were analyzed on a 2 % agarose gel stained with ethidium bromide. Gel quantifications were performed with the Adobe Photoshop® software.

### CRISPR live-cell imaging

Three specific sgRNAs for each tested locus were designed using the Broad Institute sgRNA Design Tool (http://www.broadinstitute.org/rnai/public/analysis-tools/sgrna-design-v1)^70^ and were cloned into vectors expressing either three MS2, three PP7 or six Puf1 motifs (Addgene 68426, 68424)^71^. The sgRNA sequences are listed in Supplementary Table 4. Next, using Lipofectamine 3000 (ThermoFisher Scientific, L3000008), pools of the three sgRNAs for each targeted locus (375 ng), dCas9 (Addgene 68416, 625 ng), and vectors expressing the corresponding RNA-binding proteins fused to fluorescent proteins (MS2-mVenus, PP7-mCherry, Pum1-iRFP670, each 500 ng) were transfected into MEFs and plated on LabTek v1 glass chamber slides. After 48 h of incubation, FluoroBrite™ DMEM Media (ThermoFisher Scientific, A1896701), and 1 drop NucBlue® Live ReadyProbes® Reagent (ThermoFisher Scientific R37605) were added to stain the nuclei. Images were acquired from more than 80 live cells (95 for wild type, 84 for *Firre* KO cells) by using the LSM880 with Airyscan (Zeiss) microscope, equipped with the oil immersion objective Plan-Apochromat × 63/1.4 oil DIC M27 at the Harvard Center for Biological Imaging (HCBI). Raw images were processed in ZEN (blue edition, Zeiss) and colocalization was assigned when either merged or overlapping signals (< 50 nm distance) occurred. In contrast, distinctly separated signals (> 50 nm distance) indicated no colocalization.

### Data availability

Sequencing data have been deposited in the Gene Expression Omnibus under the accession number GSE98632. All other data are available from the corresponding authors upon reasonable request.

## Electronic supplementary material

Supplementary Information(PDF 26007 kb)

## References

[1] Nora EP (2012). Spatial partitioning of the regulatory landscape of the X-inactivation centre. Nature.

[2] Dixon JR (2012). Topological domains in mammalian genomes identified by analysis of chromatin interactions. Nature.

[3] Grubert F (2015). Genetic control of chromatin states in humans involves local and distal chromosomal interactions. Cell.

[4] Pope BD (2014). Topologically associating domains are stable units of replication-timing regulation. Nature.

[5] Norton HK, Phillips-Cremins JE (2017). Crossed wires: 3D genome misfolding in human disease. J. Cell. Biol..

[6] Phillips JE, Corces VG (2009). CTCF: master weaver of the genome. Cell.

[7] Fudenberg G (2016). Formation of chromosomal domains by loop extrusion. Cell Rep..

[8] Guo Y (2015). CRISPR inversion of CTCF sites alters genome topology and enhancer/promoter function. Cell.

[9] Lupianez DG (2015). Disruptions of topological chromatin domains cause pathogenic rewiring of gene-enhancer interactions. Cell.

[10] Ong CT, Corces VG (2014). CTCF: an architectural protein bridging genome topology and function. Nat. Rev. Genet..

[11] Phillips-Cremins JE (2013). Architectural protein subclasses shape 3D organization of genomes during lineage commitment. Cell.

[12] Rao SS (2014). A 3D map of the human genome at kilobase resolution reveals principles of chromatin looping. Cell.

[13] Sanborn AL (2015). Chromatin extrusion explains key features of loop and domain formation in wild-type and engineered genomes. Proc. Natl Acad. Sci. USA.

[14] Vietri Rudan M (2015). Comparative Hi-C reveals that CTCF underlies evolution of chromosomal domain architecture. Cell Rep..

[15] Vietri Rudan M, Hadjur S (2015). Genetic tailors: CTCF and cohesin shape the genome during evolution. Trends Genet..

[16] Nora EP (2017). Targeted degradation of CTCF decouples local insulation of chromosome domains from genomic compartmentalization. Cell.

[17] Franke M (2016). Formation of new chromatin domains determines pathogenicity of genomic duplications. Nature.

[18] Rodriguez-Carballo E (2017). The HoxD cluster is a dynamic and resilient TAD boundary controlling the segregation of antagonistic regulatory landscapes. Genes Dev..

[19] Hacisuleyman E (2014). Topological organization of multichromosomal regions by the long intergenic noncoding RNA Firre. Nat. Struct. Mol. Biol..

[20] Hacisuleyman E, Shukla CJ, Weiner CL, Rinn JL (2016). Function and evolution of local repeats in the Firre locus. Nat. Commun..

[21] Deng X (2015). Bipartite structure of the inactive mouse X chromosome. Genome Biol..

[22] Giorgetti L (2016). Structural organization of the inactive X chromosome in the mouse. Nature.

[23] Darrow EM (2016). Deletion of DXZ4 on the human inactive X chromosome alters higher-order genome architecture. Proc. Natl Acad. Sci. USA.

[24] da Rocha ST, Heard E (2017). Novel players in X inactivation: insights into Xist-mediated gene silencing and chromosome conformation. Nat. Struct. Mol. Biol..

[25] Jegu T, Aeby E, Lee JT (2017). The X chromosome in space. Nat. Rev. Genet..

[26] Bonora, G. et al. Orientation-dependent Dxz4 contacts shape the 3D structure of the inactive X chromosome. Preprint at https://www.biorxiv.org/content/early/2017/12/15/165340 (2017). 10.1038/s41467-018-03694-y PMC5899087 29654302

[27] Engreitz JM, Ollikainen N, Guttman M (2016). Long non-coding RNAs: spatial amplifiers that control nuclear structure and gene expression. Nat. Rev. Mol. Cell. Biol..

[28] Rinn J, Guttman M (2014). RNA Function. RNA and dynamic nuclear organization. Science.

[29] Tan JY (2017). cis-Acting complex-trait-associated lincRNA expression correlates with modulation of chromosomal architecture. Cell Rep..

[30] Amaral, R. P. et al. Genomic positional conservation identifies topological anchor point RNAs linked to developmental loci. *Genome Biol.* **19, **32 (2018). 10.1186/s13059-018-1405-5 PMC5853149 29540241

[31] Kung JT (2015). Locus-specific targeting to the X chromosome revealed by the RNA interactome of CTCF. Mol. Cell..

[32] Mele M, Rinn JL (2016). “Cat’s Cradling” the 3D genome by the act of LncRNA transcription. Mol. Cell..

[33] Andergassen, D. et al. Mapping the mouse Allelome reveals tissue-specific regulation of allelic expression. *eL**ife* **6**, e25125 (2017). 10.7554/eLife.25125 PMC5555720 28806168

[34] Maass PG (2018). Spatiotemporal allele organization by allele-specific CRISPR live-cell imaging (SNP-CLING). Nat. Struct. Mol. Biol..

[35] Naumova N, Smith EM, Zhan Y, Dekker J (2012). Analysis of long-range chromatin interactions using chromosome conformation capture. Methods.

[36] de Wit E (2015). CTCF binding polarity determines chromatin looping. Mol. Cell..

[37] Goloborodko, A., Imakaev, M. V., Marko, J. F. & Mirny, L. Compaction and segregation of sister chromatids via active loop extrusion. *eLife* **5**, e14864 (2016). 10.7554/eLife.14864 PMC4914367 27192037

[38] Rodriguez-Carballo E (2017). The HoxD cluster is a dynamic and resilient TAD boundary controlling the segregation of antagonistic regulatory landscapes. Genes Dev..

[39] Narendra V (2015). CTCF establishes discrete functional chromatin domains at the Hox clusters during differentiation. Science.

[40] Rao SSP (2017). Cohesin loss eliminates all loop domains. Cell.

[41] Schwarzer W (2017). Two independent modes of chromatin organization revealed by cohesin removal. Nature.

[42] Beagan JA (2017). YY1 and CTCF orchestrate a 3D chromatin looping switch during early neural lineage commitment. Genome Res..

[43] Weintraub AS (2017). YY1 is a structural regulator of enhancer-promoter loops. Cell.

[44] Rowley MJ (2017). Evolutionarily conserved principles predict 3D chromatin organization. Mol. Cell..

[45] Jeong, M. et al. A cell type-specific class of chromatin loops anchored at large DNA methylation nadirs. Preprint at https://www.biorxiv.org/content/early/2017/11/09/212928.1 (2017).

[46] Barutcu AR (2016). SMARCA4 regulates gene expression and higher-order chromatin structure in proliferating mammary epithelial cells. Genome Res..

[47] Barutcu AR, Lian JB, Stein JL, Stein GS, Imbalzano AN (2017). The connection between BRG1, CTCF and topoisomerases at TAD boundaries. Nucleus.

[48] Uuskula-Reimand L (2016). Topoisomerase II beta interacts with cohesin and CTCF at topological domain borders. Genome Biol..

[49] Yu M, Ren B (2017). The three-dimensional organization of mammalian genomes. Annu. Rev. Cell. Dev. Biol..

[50] Ou, H. D. et al. ChromEMT: Visualizing 3D chromatin structure and compaction in interphase and mitotic cells. *Science* **357**, eaag0025 (2017). 10.1126/science.aag0025 PMC5646685 28751582

[51] Dixon JR, Gorkin DU, Ren B (2016). Chromatin domains: the unit of chromosome organization. Mol. Cell..

[52] Durand NC (2016). Juicebox provides a visualization system for Hi-C contact maps with unlimited zoom. Cell Syst..

[53] Durand NC (2016). Juicer provides a one-click system for analyzing loop-resolution Hi-C experiments. Cell Syst..

[54] Servant N (2015). HiC-Pro: an optimized and flexible pipeline for Hi-C data processing. Genome Biol..

[55] Jung YH (2017). Chromatin states in mouse sperm correlate with embryonic and adult regulatory landscapes. Cell Rep..

[56] Wang H (2012). Widespread plasticity in CTCF occupancy linked to DNA methylation. Genome Res..

[57] Consortium EP (2012). An integrated encyclopedia of DNA elements in the human genome. Nature.

[58] Niakan KK, Schrode N, Cho LT, Hadjantonakis AK (2013). Derivation of extraembryonic endoderm stem (XEN) cells from mouse embryos and embryonic stem cells. Nat. Protoc..

[59] Langmead B, Salzberg SL (2012). Fast gapped-read alignment with Bowtie 2. Nat. Methods.

[60] Li B, Dewey CN (2011). RSEM: accurate transcript quantification from RNA-Seq data with or without a reference genome. BMC Bioinformatics.

[61] Love MI, Huber W, Anders S (2014). Moderated estimation of fold change and dispersion for RNA-seq data with DESeq2. Genome Biol..

[62] Lee TI, Johnstone SE, Young RA (2006). Chromatin immunoprecipitation and microarray-based analysis of protein location. Nat. Protoc..

[63] Heinz S (2010). Simple combinations of lineage-determining transcription factors prime cis-regulatory elements required for macrophage and B cell identities. Mol. Cell..

[64] Grant CE, Bailey TL, Noble WS (2011). FIMO: scanning for occurrences of a given motif. Bioinformatics.

[65] Sandelin A, Alkema W, Engstrom P, Wasserman WW, Lenhard B (2004). JASPAR: an open-access database for eukaryotic transcription factor binding profiles. Nucleic Acids Res..

[66] Belaghzal H, Dekker J, Gibcus JHHiC (2017). 2.0: An optimized Hi-C procedure for high-resolution genome-wide mapping of chromosome conformation. Methods.

[67] Keane TM (2011). Mouse genomic variation and its effect on phenotypes and gene regulation. Nature.

[68] Quinlan AR, Hall IM (2010). BEDTools: a flexible suite of utilities for comparing genomic features. Bioinformatics.

[69] Crane E (2015). Condensin-driven remodelling of X chromosome topology during dosage compensation. Nature.

[70] Doench JG (2014). Rational design of highly active sgRNAs for CRISPR-Cas9-mediated gene inactivation. Nat. Biotechnol..

[71] Shechner DM, Hacisuleyman E, Younger ST, Rinn JL (2015). Multiplexable, locus-specific targeting of long RNAs with CRISPR-Display. Nat. Methods.

