# Supplementary material for: A TAD boundary is preserved upon deletion of the CTCF-rich Firre locus
Source: Nat Commun. 2018 Apr 13;9:1444. doi: 10.1038/s41467-018-03614-0 (PMC5899154; doi:10.1038/s41467-018-03614-0)
Supplement: Supplementary file 1 — Supplementary Information(PDF 26007 kb) [file 41467_2018_3614_MOESM1_ESM.pdf]

Supplementary Information:

A TAD boundary is preserved upon deletion of the CTCF-rich *Firre* locus

Barutcu *et al*.

Contents:

Supplementary Figures 1-11
Supplementary Tables 1-4

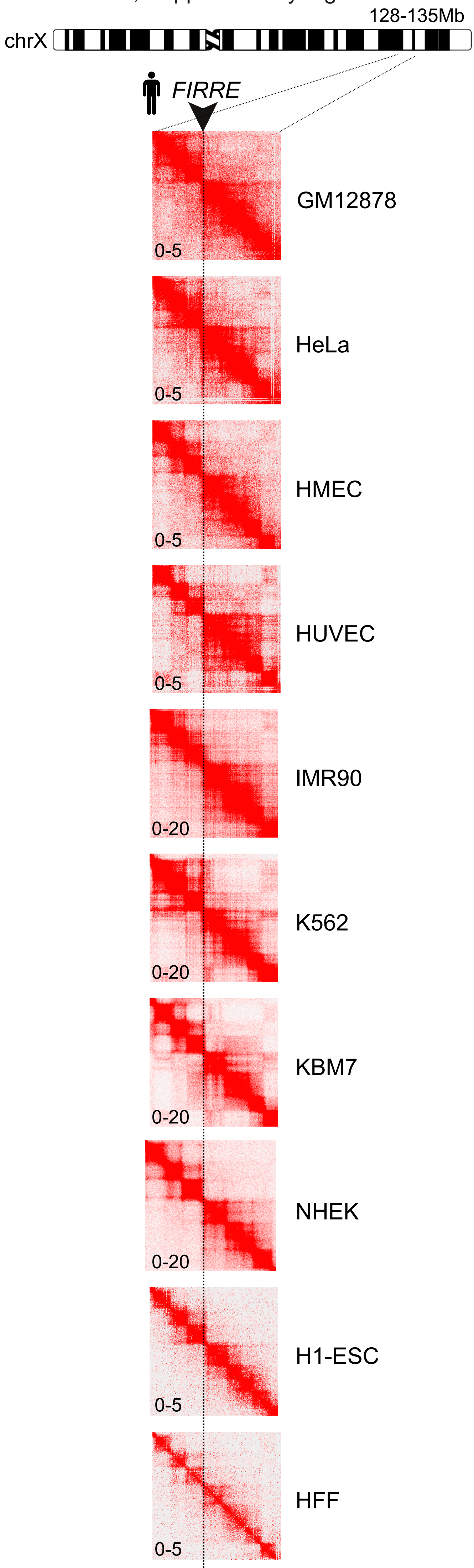

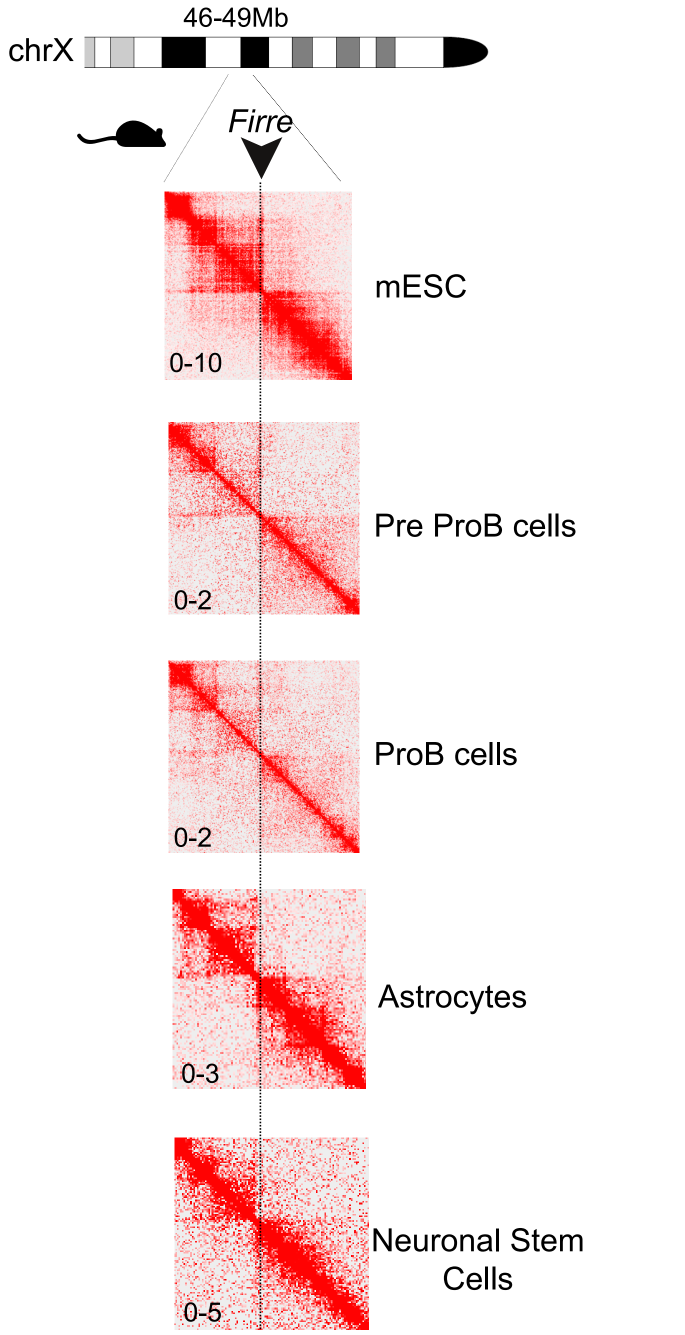

Supplementary Figure 1 – *FIRRE* / *Firre* lncRNA gene is consistently located at a TAD boundary in multiple human and mouse cell types. Hi-C interaction heatmaps, extracted with the Juicebox software, of the FIRRE / *Firre* locus in multiple human cell types. The dashed line designates the FIRRE / *Firre* locus, which consistently lies at a TAD boundary.

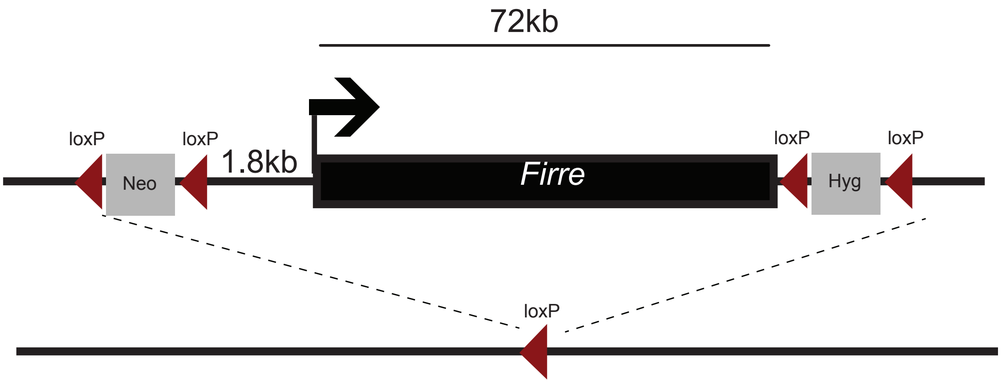

Supplementary Figure 2 – Deletion strategy of the *Firre* lncRNA. Upon *Firre* deletion, the entire *Firre* gene, the promoter region and 1.8kb of the 3' end flanking sequences are removed (see Methods) (mm9, chrX:47908463-47990293).

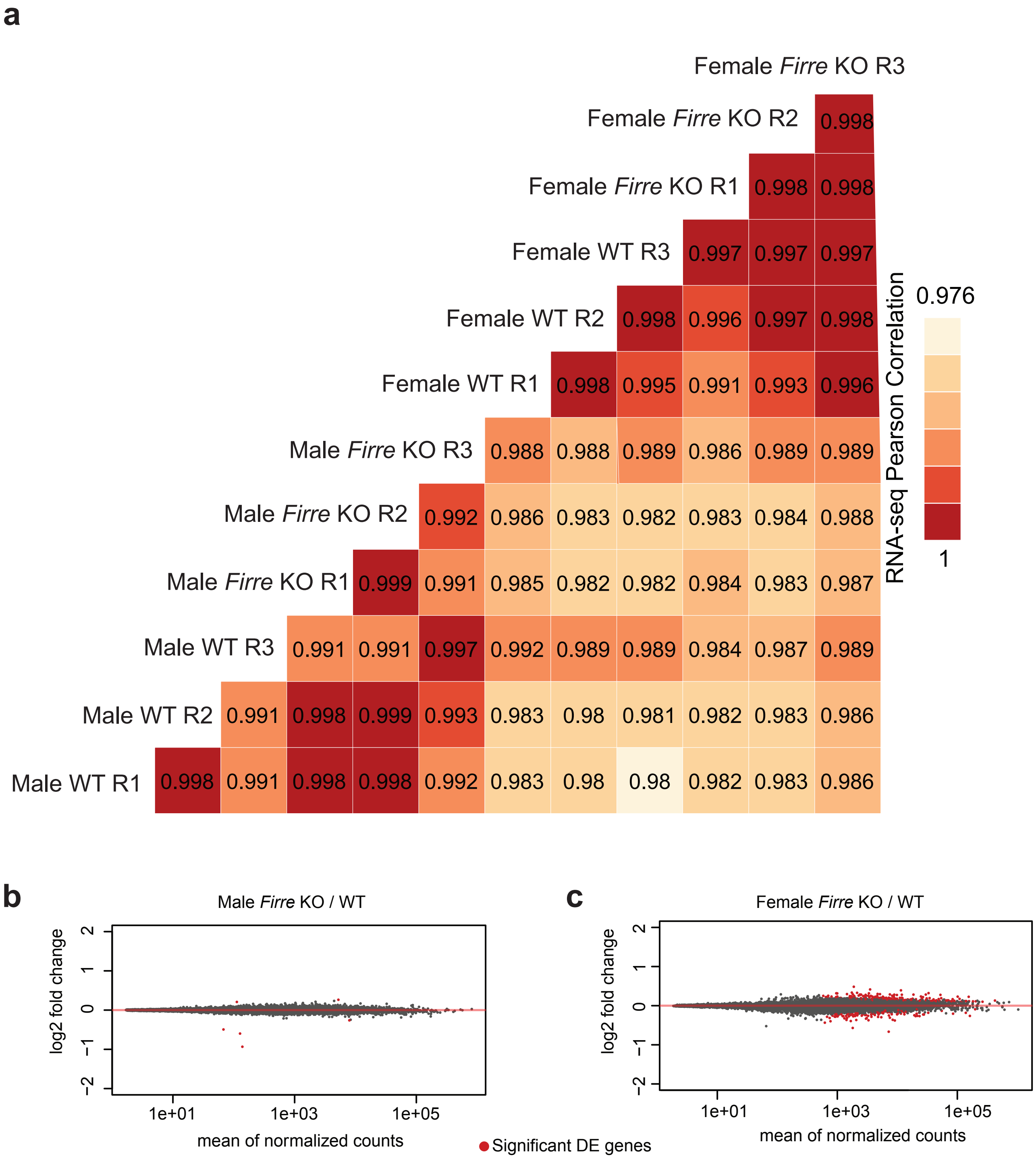

Supplementary Figure 3 – RNA-seq analysis of *Firre* KO and wildtype male and female MEFs. a) Pearson correlation matrix showing the reproducibility between each RNAseq replicate. **b-c)** Plots showing the expression levels of the genes as a function of *Firre* KO vs. WT log2 fold change in **b)** male cells, and **c)** in female cells. *Firre* shows a higher number of significantly down-regulated genes in female samples compared to males.

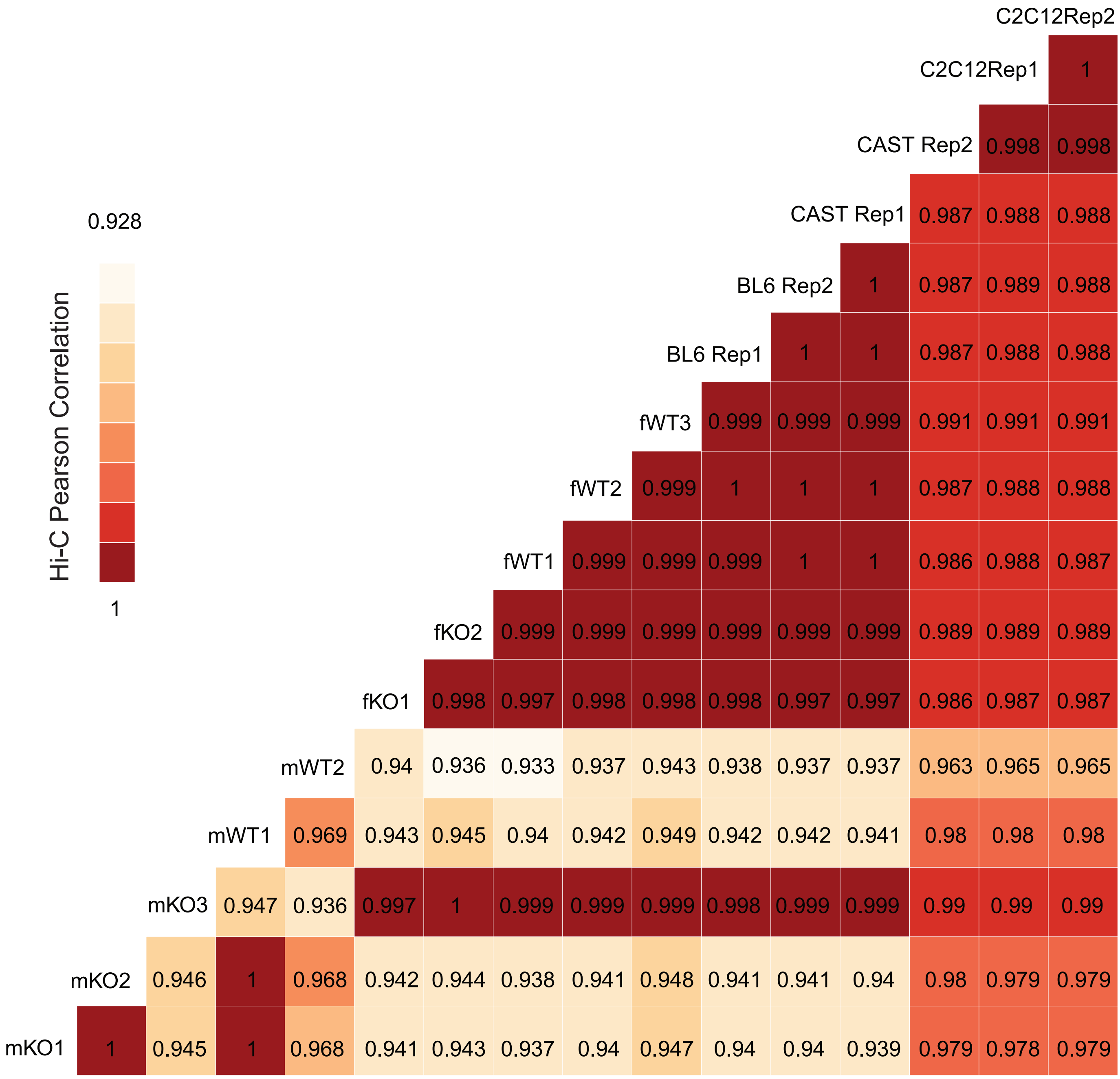

Supplementary Figure 4 – Hi-C Correlations. Pearson correlation matrix showing the reproducibility between each Hi-C replicate for male, female and allele specific MEFs (C57BL6 x CAST), as well as C2C12 cells.

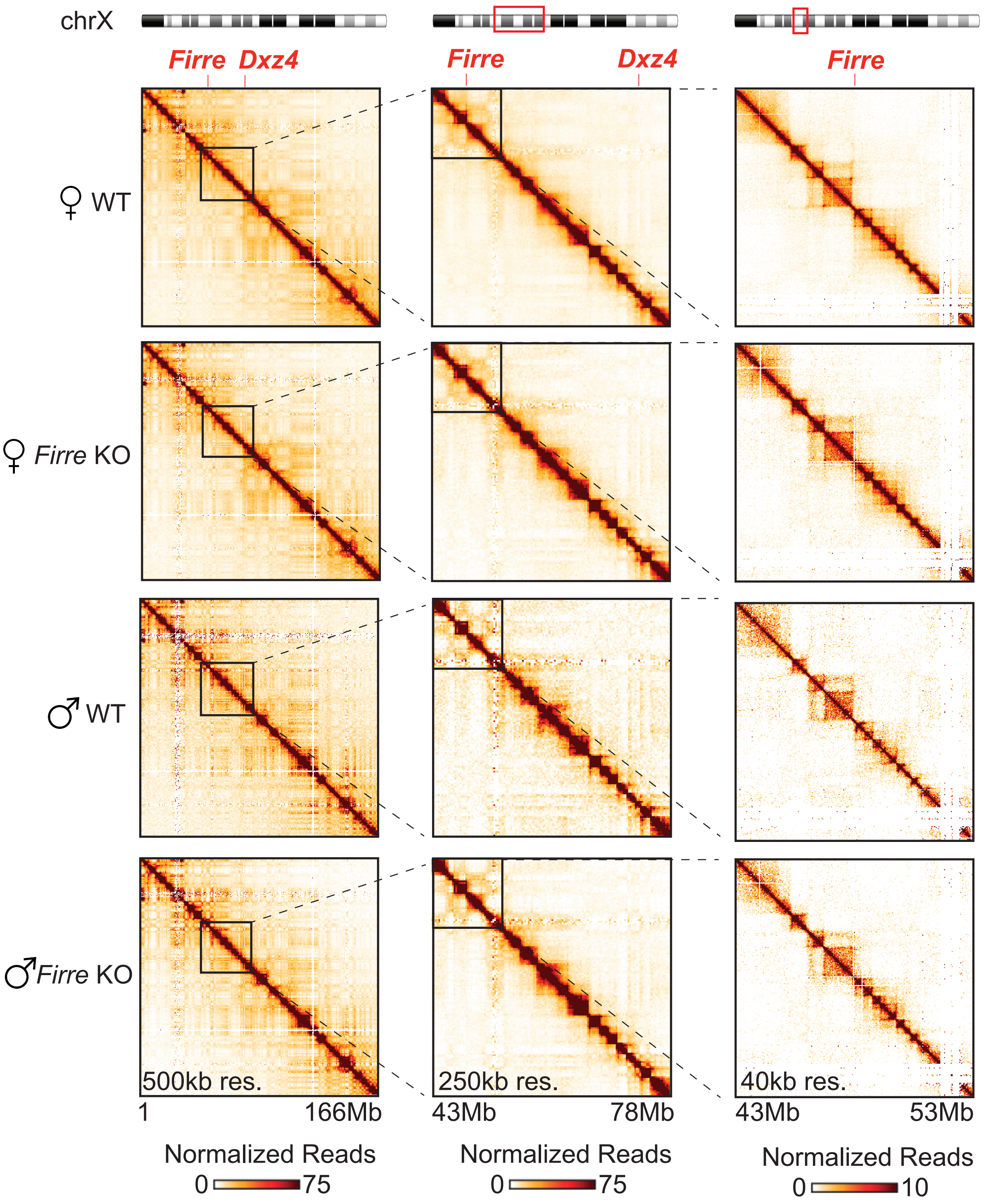

Supplementary Figure 5 - Interaction heatmaps of the MEF Hi-C data. Heatmaps for female and male wildtype and *Firre* KO combined MEF Hi-C samples for chromosome X at 500kb resolution (left), with a zoom in between 43-78Mb at 250kb resolution (middle), and between 43-53Mb (Firre locus) at 40kb resolution.

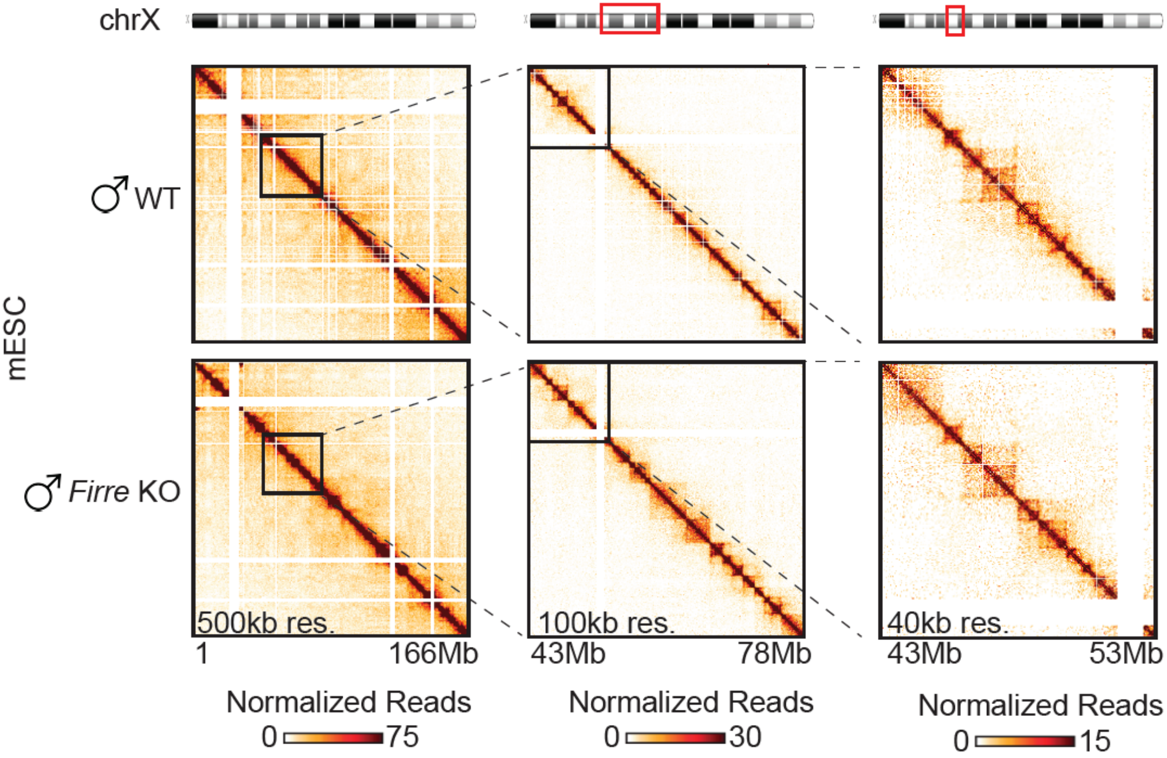

Supplementary Figure 6 - Hi-C data of wildtype and *Firre* KO mESCs. Heatmaps for female and male wildtype and *Firre* KO combined mESC Hi-C samples for chromosome X at 500kb resolution (left), with a zoom in between 43-78Mb at 100kb resolution (middle), and between 43-53Mb (*Firre* locus) at 40kb resolution.

Barutcu *et al.*, Supplementary Figure 7

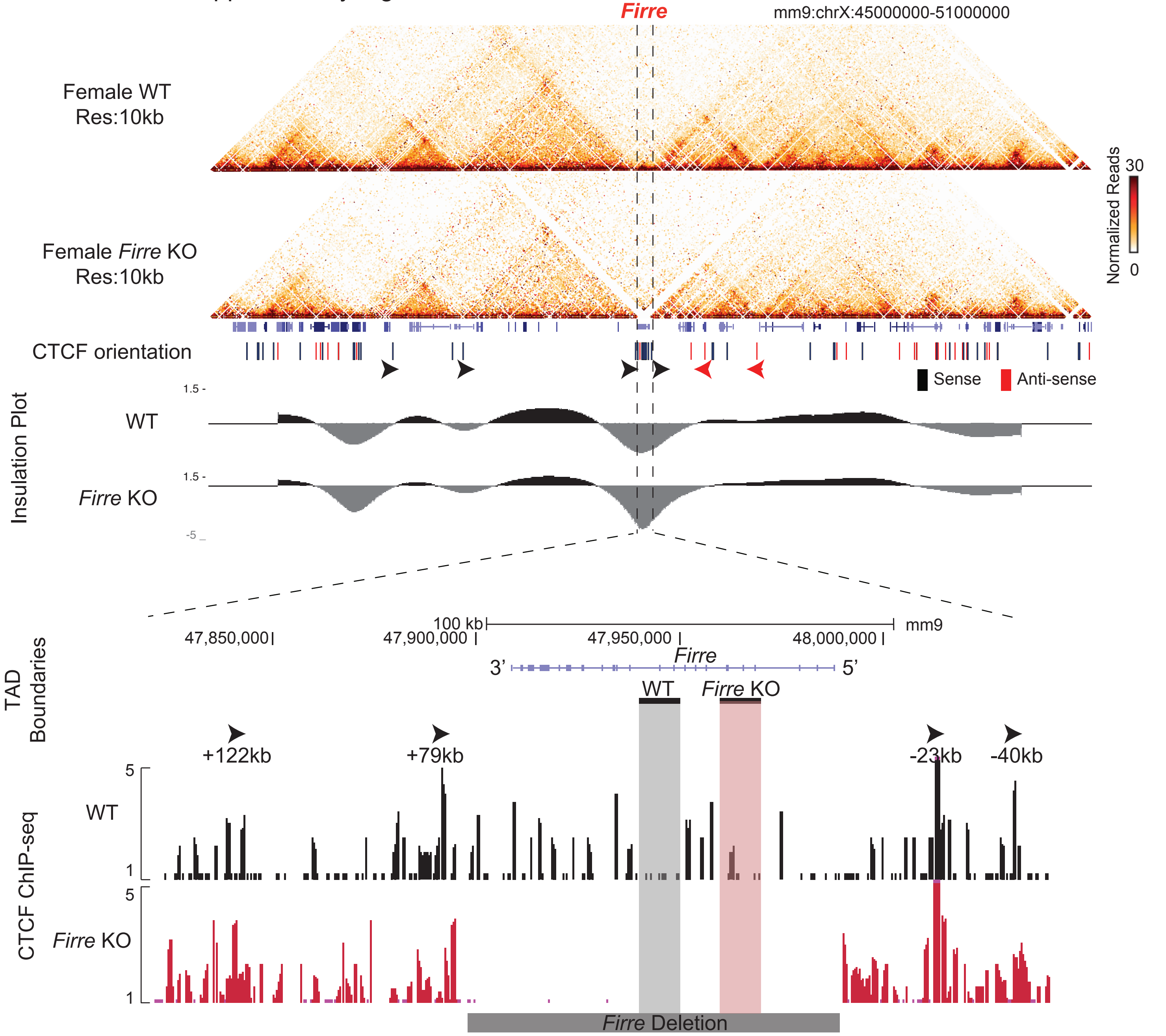

Supplementary Figure 7 – Surrounding CTCF binding sites are not located on the *Firre* TAD boundary. 10kb resolution Hi-C heatmaps of female WT and Firre KO samples, the UCSC gene track and the CTCF orientation is shown, highlighting the orientation of the CTCF sites surrounding *Firre* and the neighboring boundaries. Below, a zoom-in view showing the TAD boundaries and the CTCF ChIP-seq signal track from wildtype and *Firre* KO MEFs showing the four surrounding CTCF sites located at +122kb, +79kb, -23kb and -40kb of the *Firre* transcriptional start site (TSS), outside of the *Firre* TAD boundary (highlighted).

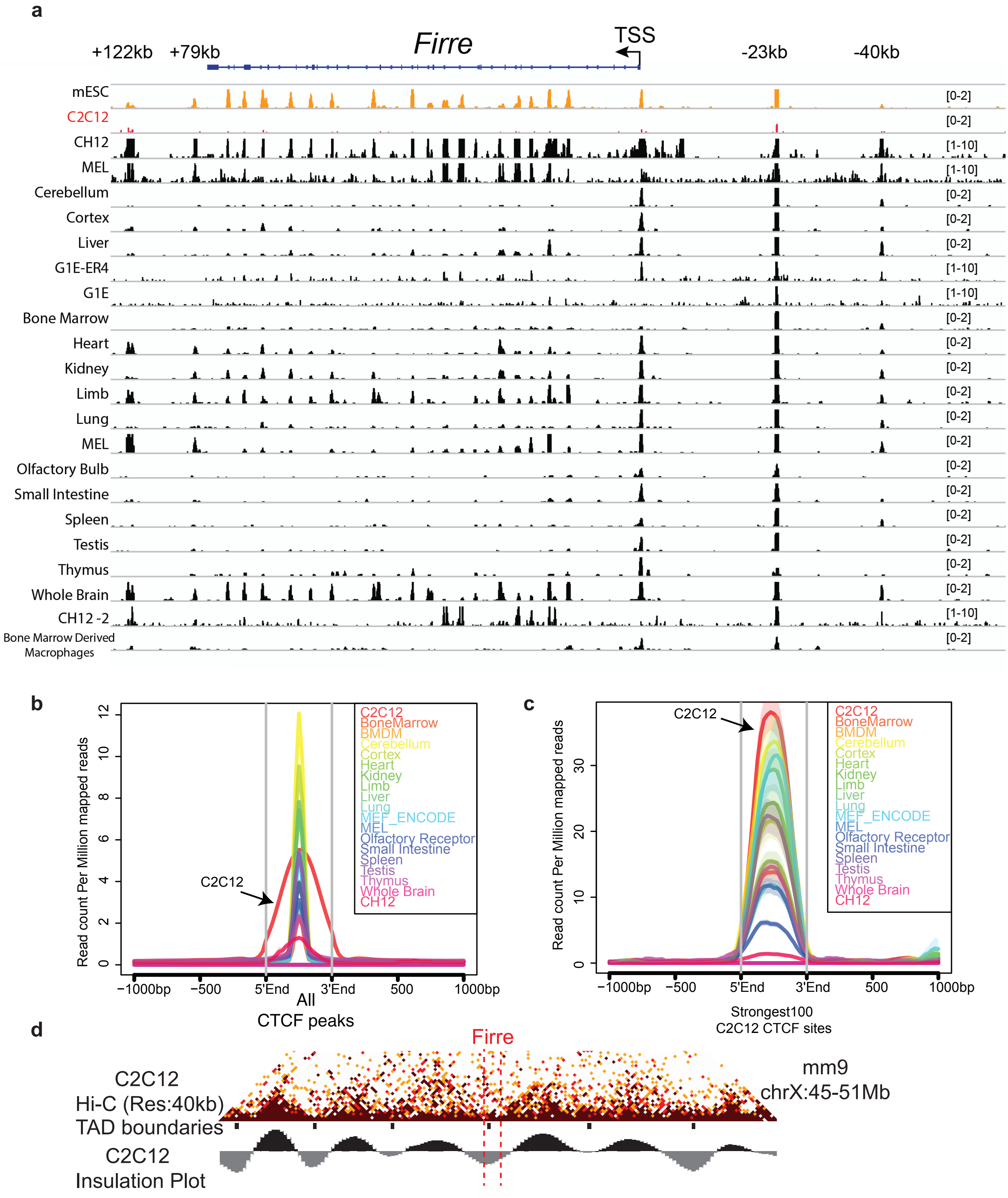

Supplementary Figure 8 – C2C12 cells shows reduced CTCF binding at surrounding sites. The preservation of the *Firre* TAD boundary is not compensated by the surrounding CTCF sites. **a)** IGV browser track showing the CTCF binding neighboring the Firre locus in multiple mouse cell types. 3 of the 4 neighboring CTCF peaks showed variable binding across the cell types, indicating that these sites cannot account for the TAD boundary formation since the boundary can form in their absence. C2C12 cells show reduced CTCF binding throughout the region with minimal signal at the -23kb position. **b)** ChIP-seq signal intensity for C2C12 and the ENCODE CTCF datasets centered on all CTCF peaks for each cell type. **c)** Average signal profile for the ENCODE CTCF data centered strongest 100 C2C12 peaks, indicating that the reduced signal at -23kb is not due to a bias in the C2C12 dataset. **d)** C2C12 Hi-C interaction heatmap at 40kb resolution showing +/- 5 Mb of the *Firre* gene locus (mm9 chrX:45-51Mb), with the TAD boundary and insulation track below. Less intense binding of the +122kb, +79kb and -40kb CTCF sites cannot fully explain the establishment or maintenance of the Firre TAD boundary.

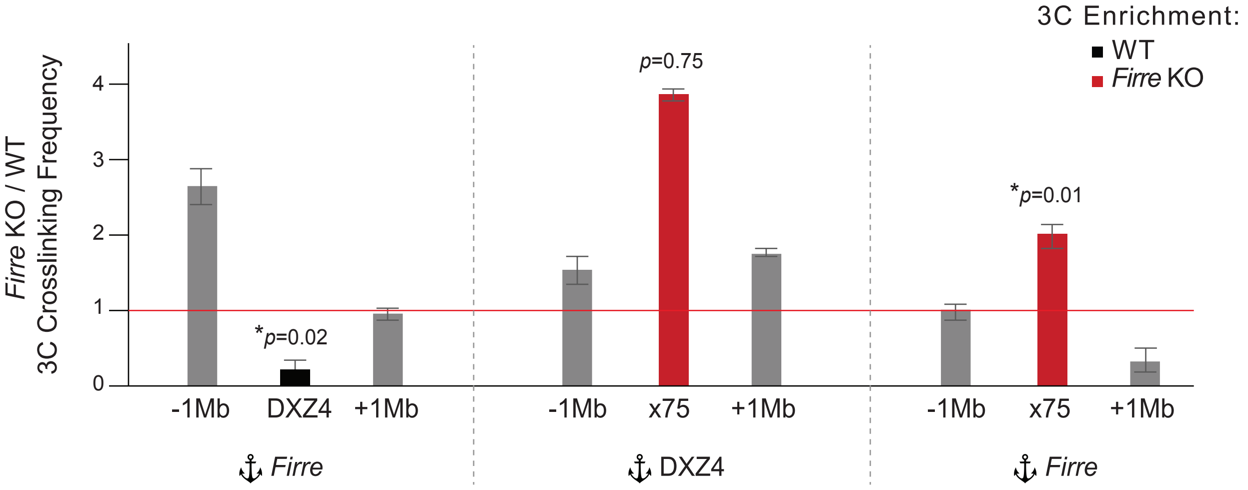

Supplementary Figure 9 – Chromosome conformation capture (3C) analysis of superloop interactions validate Hi-C and live-cell imaging results. Female *Firre* KO versus wildtype 3C interaction frequency ratios between *Firre*, DXZ4 and x75, along with 1Mb surrounding regions. Interaction frequencies less than 1 indicates 3C enrichment in the wildtype sample. The anchor primers are indicated below each section.

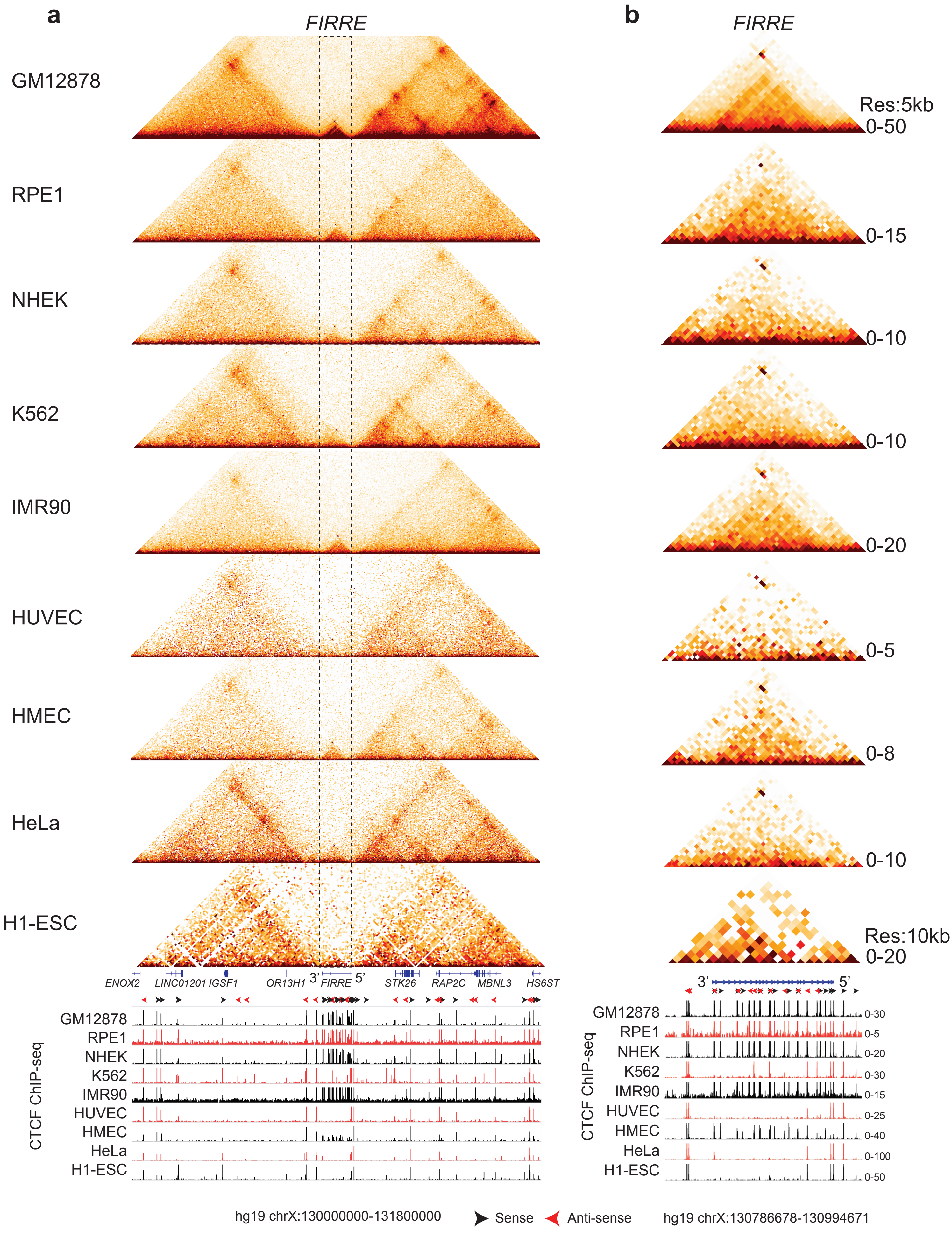

Supplementary Figure 10 – High resolution interaction topology of the *FIRRE* locus in human samples. a) Hi-C interaction heatmaps showing the neighboring TADs of the *FIRRE* locus in multiple cell types. The CTCF binding and orientation is included below. In all cell types, *FIRRE* seems to be making a circuar looping structure, a feature not so prevalent in mouse (Supplementary Figure 11). **b)** A zoom-in of the interaction profile of *FIRRE* gene with CTCF binding and orientations. The presence of a circular formation can be observed by a dark interacting bin between the 3' and 5' end of *FIRRE*. The H1-ES cells does not appear to be forming such a structure.

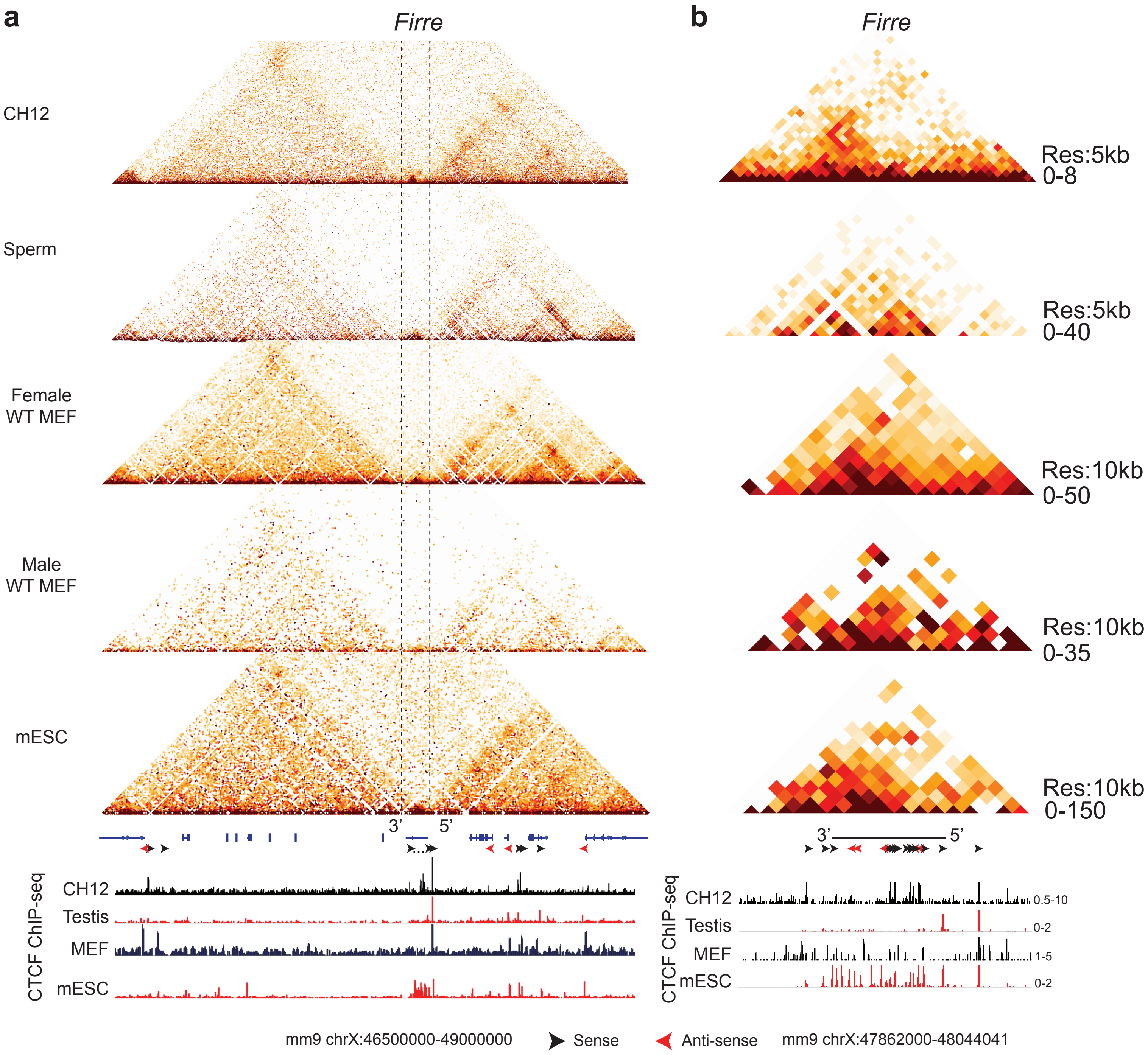

Supplementary Figure 11 – High resolution interaction topology of the *Firre* locus in mouse samples. a) Hi-C interaction heatmaps showing the neighboring TADs of the *Firre* locus in CH12 cells, male and female MEFs and mESCs. The CTCF binding and orientation is included below. The binding of CTCF in convergent orientation, although can explain the presence of the 5' TAD, does not explain the 3' TAD formation (sense-sense orientation). **b)** A zoom-in of the interaction profile of *Firre* gene with CTCF binding and orientations. There does not seem to be a prevalent circular formation as in the case of human samples (Supplementary Figure 10).

Supplementary Table 1 - Read statistics for Hi-C samples

Hi-C Sample	Raw read pairs	Uniquely Mapped read pairs	Valid interactions
Male MEF WT-R1	114,678,463	95,404,889	69,318,730
Male MEF WT-R2	86,882,511	76,518,504	53,749,827
Male MEF WT-R3	271,487,347	165,513,925	138,321,004
Male MEF FIRRE KO-R1	106,726,083	85,117,692	54,302,424
Male MEF FIRRE KO-R2	91,092,126	72,591,309	45,617,460
Female MEF WT-R1	300,118,001	276,153,109	201,186,013
Female MEF WT-R2	264,381,469	222,535,419	156,227,921
Female MEF WT-R3	53,809,968	51,401,310	33,435,762
Female MEF FIRRE KO-R1	200,075,316	185,816,580	134,063,833
Female MEF FIRRE KO-R2	134,741,024	125,441,114	88,843,107
Female MEF FIRRE KO-R3	124,199,684	28,072,401	24,603,248
Male MEF WT Combined	473,048,321	288,582,482	213,998,225
Male MEF FIRRE KO Combined	197,818,209	99,919,884	79,967,990
Female MEF WT Combined	726,169,857	457,968,046	340,402,145
Female MEF FIRRE KO Combined	459,016,024	256,742,380	186,778,656
Minus DOX tg24/rTTA -/y	256,039,805	126,530,427	108,778,333
Plus DOX tg24/rTTA -/y	179,119,831	82,450,869	70,630,033
Firre KO mESC Combined	105,146,120	95,700,113	48,736,700
WT N3 mESC Combined	321,517,882	108,590,279	68,002,698

Allele-specific Hi-C Samples	Raw read pairs	Uniquely Mapped read pairs	Interactions for mm9
Female C57BL6 FIRRE KO x CAST WT Rep1	155,422,998	90,816,396	86,803,615
Female C57BL6 FIRRE KO x CAST WT Rep2	222,461,549	122,533,811	115,605,400
Female C57BL6 FIRRE KO x CAST WT Combined	377,884,547	213,350,207	144,528,591
Male C57BL6 FIRRE KO x CAST WT Rep1	75,147,577	42,035,680	31,113,184
Male C57BL6 FIRRE KO x CAST WT Rep2	89,840,746	49,792,476	42,642,939
Male C57BL6 FIRRE KO x CAST WT Combined	164,988,323	91,828,156	73,756,123

Allele-specific Hi-C Samples	Interactions for C57BL6	Interactions for CAST
Female C57BL6 FIRRE KO x CAST WT Rep1	26,846,457	24,206,598
Female C57BL6 FIRRE KO x CAST WT Rep2	38,266,250	32,323,800
Female C57BL6 FIRRE KO x CAST WT Combined	46,249,797	40,590,782
Male C57BL6 FIRRE KO x CAST WT Rep1	11,093,395	9,602,935
Male C57BL6 FIRRE KO x CAST WT Rep2	15,568,642	13,323,808
Male C57BL6 FIRRE KO x CAST WT Combined	24,873,358	21,388,725

Supplementary Table 2: qPCR and Genotyping Primers

F_Firre-1_Ex	TCACAATGGGCTGGGTATTCTC
R_Firre-1_Ex	CCTGGGTCCTCTATAAAAGCAACAG
F_Sry	TTGTCTAGAGAGCATGGAGGGCCATGTCAA
R_Sry	CCACTCCTCTGTGACACTTTAGCCCTCCGA
F_FirreWT_2	GGAGGAGTGCTGCTTACTGG
R_FirreWT_2	TCTGTGAGCCACCTGAAATG
F_TRE	TACCACTCCCTATCAGTGA
R_Firre	CGGCTTCATCTTCAGTCCTC
F_ActB_qPCR	GCTGTATTCCCCTCCATCGTG
R_ActB_qPCR	CACGGTTGGCCTTAGGGTTCAG
F_Gapdh_qPCR	GGTGAAGGTCGGTGTGAACG
R_Gapdh_qPCR	CTCGCTCCTGGAAGATGGTG
F1-Firre1.49_qRTPCR	AAATCCGAGGACAGTCGAGC
R1-Firre1.49_qRTPCR	CCGTGGCTGGTGACTTTTTG

Supplementary Table 3 - List of 3C primers

DXZ4	ACAATGGCTTGTGCTGTAAGATT
DXZ4 plus 1Mb	AAGTGGAGCATGTGTCCTTCTTA
DXZ4 minus1Mb	CCAGTAAGCTAAGGCTCAACAGA
x75_minus1Mb	TCTGTAATCTGCACTCTCACCTG
x75	GTCTTATTGACGGTGTCTTTTGC
x75_plus1Mb	CCATTTCCTAAGTGACATGCTTC
Firre_minus1Mb	AATAGATCCTCTAGGGTGCTTGG
Firre	TTTCATTTCTGTCTGGATGACCT
Firre_plus1Mb	GACTTAGCTTTGGAAAGCACTGA

Supplementary Table 4 - List of CRISPR/Cas9 live-cell imaging sgRNAs

mFirre_1FW	CACCG TACCCTCAGTGTCAGCACTG
mFirre_2FW	CACCG TACTCAGGGTATGCCCACTG
mFirre_3FW	CACCG AGCAATAAAACTGTAAGACA
mDXZ4_1FW	CACCG TCCTATGAGAGAGAATTGTG
mDXZ4_2FW	CACCG TGAGTGGAATAAGTCTCCCA
mDXZ4_3FW	CACC GTGTGCAGCTGAACACGTCG
mx75_1FW	CACC G TTTGTACGGAACTTCACCCG
mx75_2FW	CACC GGACGCTGGGACTAAGTCAG
mx75_3FW	CACC G ATCTCCGGAGCTAAACGGTG
